# LNC297 promotes BMSCs differentiation and alleviates BHBA-induced inhibition through the miR-145/GAS7 axis

**DOI:** 10.1371/journal.pone.0354035

**Published:** 2026-07-22

**Authors:** Tao Tang, Jing Zhou, Xianbo Jia, Jiahao Shao, Meigui Wang, Siqi Xia, Shuai Chen, Wenqiang Sun, Jie Wang, Song-jia Lai

**Affiliations:** 1 College of Animal Science and Technology, Sichuan Agricultural University, Chengdu, Sichuan, China; 2 Farm Animal Genetic Resources Exploration and Innovation Key Laboratory of Sichuan Province, Sichuan Agricultural University, Chengdu, Sichuan, China; 3 College of Animal Science and Technology, Southwest University, Chongqing, Rongchang, China; 4 Hong Feng Dairy Cow Breeding Co., Ltd., Mianyang, Anzhou District, China; University of Vermont, UNITED STATES OF AMERICA

## Abstract

Ketosis in dairy cows is characterized by elevated circulating β-hydroxybutyrate (BHBA) and profound alterations in whole-body energy metabolism. As a major peripheral tissue responsible for energy consumption and substrate utilization, skeletal muscle is continuously exposed to increased BHBA during ketosis and may use BHBA as an alternative energy substrate. However, the regulatory mechanism by which BHBA affects skeletal muscle differentiation remains unclear.This study used bovine skeletal muscle satellite cells (BMSCs) to investigate the effects of BHBA on the differentiation of BMSCs and to explore the regulatory roles of LNC297, miR-145, and GAS7 in this process. The results of dual-luciferase reporter gene assays and miRNA pull-down experiments verified the targeting relationship between LNC297, miR-145, and GAS7. BHBA inhibits the differentiation of BMSCs in a dose-dependent manner. Both LNC297 and GAS7 promote the differentiation of BMSCs and attenuate the inhibitory effect of BHBA. In contrast, miR-145 inhibits BMSCs differentiation and enhances the inhibitory effect of BHBA. Mechanistically, LNC297 acts as a competing endogenous RNA (ceRNA) or molecular sponge for miR-145, thereby upregulating GAS7 expression and promoting its differentiation function. LNC297 may promote muscle differentiation through the miR-145/GAS7 axis, thereby alleviating muscle damage in cows with ketosis, providing a new perspective for investigating the mechanisms of muscle development disorders.

## 1. Introduction

In late pregnancy and early postpartum, dairy cows often enter a state of negative energy balance (NEB) due to factors such as increased fetal size leading to uterine compression, hormonal changes, and insufficient energy intake [1]. At this stage, dairy cows mobilize stored body fat, which is converted into triglycerides (TG). These TGs are then broken down into non-esterified fatty acids (NEFA). When NEFAs undergo incomplete oxidation in the liver, a large amount of ketone bodies is produced and released into the bloodstream [2]. In addition to metabolic disorders or enzyme deficiencies, the formation of ketone bodies (KBs) is commonly associated with the following metabolic conditions: The first type is low carbohydrate availability. During fasting, negative energy balance, or ketosis, glucose and its metabolites—particularly oxaloacetate—are depleted. Because oxaloacetate is diverted toward gluconeogenesis, acetyl-CoA derived from fatty acid β-oxidation cannot efficiently enter the TCA cycle. Consequently, acetyl-CoA accumulates and is redirected toward ketone body synthesis rather than being fully oxidized to CO₂ and H₂O [3]. The second type is characterized by increased influx of NEFA and elevated β-oxidation. Increased lipolysis during negative energy balance elevates circulating NEFAs, enhancing hepatic uptake and β-oxidation. When the production of acetyl-CoA exceeds the oxidative capacity of the TCA cycle, the excess acetyl-CoA is converted into KBs.

KBs include acetoacetate, β-hydroxybutyrate, and acetone. When the body experiences energy deficiency, they can be used as fuel and transported to extrahepatic tissues for oxidation [4]. In dairy cows, inadequate metabolic adaptation results in excessive BHBA production from NEFA, and when blood BHBA exceeds 1.2 mM, ketosis develops [5]. BHBA might also serve as an alternative energy source to supply energy to muscles and maintain their specific physiological functions [6,7].

Recent studies have shown that the expression levels of MYH4 and MYH7 proteins in the muscle tissue of ketosis dairy cows are decreased [8]. MYH7 is a slow muscle-type myosin heavy chain (β-myosin), which, during muscle injury, is involved in the activation of satellite cells that migrate to the damaged area and differentiate into muscle fibers [9]. The expression of MYH7 is closely related to the generation of slow muscle fibers and muscle regeneration. Increased expression of MYH7 may promote the differentiation of satellite cells, particularly in the development of slow muscle fiber types [10]. MYH4 is a fast muscle fiber-type myosin heavy chain, primarily involved in rapid contraction and force generation. The coordinated action of MYH7 and MYH4 may help satellite cells adapt to the post-injury environment, selectively differentiating into the appropriate muscle fiber types, thereby aiding in muscle function recovery [11]. However, previous studies have not revealed specific changes in the differentiation of skeletal muscle satellite cells in cows with ketosis, nor whether high concentrations of BHBA affect the differentiation of BMSCs.

Long non-coding RNAs (lncRNAs) are nucleotide sequences longer than 200 base pairs. lncRNAs are involved in various biological processes, including cell proliferation, differentiation, and apoptosis [12,13]. The growth and development of skeletal muscle begin in the embryonic mesoderm. Mesenchymal stem cells derived from the embryonic mesoderm differentiate into myoblasts, which then proliferate and differentiate to form myotubes. These myotubes further mature into muscle fibers. Ultimately, the muscle fibers grow and develop into mature skeletal muscle [14,15]. This process relies on the coordinated regulation of coding genes and non-coding RNAs, such as muscle regulatory factors (MRFs) [16], paired box proteins 3/7 (Pax3/7), and the myocyte enhancer factor 2 (MEF2) family [17]. LncRNAs function as “molecular scaffolds,” “decoys,” “guiding molecules,” and “signaling molecules” to activate or repress transcription [18]. LncRNA H19 promotes the differentiation of bovine skeletal muscle satellite cells by inhibiting the Sirt1/FoxO1 pathway [19].MicroRNA(miRNA) is a small non-coding RNA molecule, 21 to 23 nucleotides in length, classified as a highly conserved class of endogenous short non-coding RNAs [20]. They mainly function by pairing with complementary sequences in the 3' UTR region of target genes, thereby promoting mRNA degradation or inhibiting its translation [21]. For example, miR-103 and miR-139 affect the differentiation of bovine skeletal muscle-derived satellite cells by regulating the gene expression of cyclin E1 (CCNE1) and dihydrofolate reductase (DHFR), respectively [22]. In addition, lncRNA might act as a competitive endogenous RNA (ceRNA) to sequester miRNA from its target mRNA and participate in the regulation of muscle development [23,24]. Previous studies have shown that lncRNA-MEG3 promotes the differentiation of bovine skeletal muscle by interacting with miRNA-135 and MEF2C, thereby enhancing the expression of genes such as MHC and MYOG [25].

Our previous sequencing data indicated that, during the early stages of calving, muscle fiber formation in dairy cows with ketosis can be impaired. We also observed an increase in the expression of an unnamed lncRNA, MSTRG29792.1 (LNC297), a decrease in the expression of a newly identified miRNA (miR-145), and a reduction in the expression of the downstream target gene GAS7. However, it is currently unclear how BHBA affects the differentiation of BMSCs, as well as whether LNC297, miR-145, and *GAS7* can regulate skeletal muscle cell differentiation and the underlying regulatory mechanisms involved. Additionally, the potential regulation of muscle function under the influence of BHBA is not well understood. In this study, we found that BHBA inhibits BMSCs differentiation, LNC297 acts as a molecular sponge for miR-145, inhibiting its expression and promoting the expression of GAS7, thus enhancing BMSCs differentiation and alleviating the differentiation inhibition induced by BHBA. This could serve as a potential target for the treatment of ketosis in dairy cows.

## 2. Materials and methods

### 2.1. Ethics statement

All animal experiments in the present study involving animals were performed under the direction of the Institutional Animal Care and Use Committee from the College of Animal Science and Technology, Sichuan Agricultural University, China. (Certification No. SYXK2019−187; Approval time: 2019/1/29). The BMSCs were derived from the right hindlimb muscle tissue of a newly born Chinese Holstein dairy calf. Calves were euthanized at a predetermined experimental endpoint for skeletal muscle collection. Humane endpoints were also established to ensure early euthanasia if animals showed signs of severe pain or distress. Calves were euthanized at a predetermined experimental endpoint for skeletal muscle tissue collection. The pre-determined experiment refers to newborn calves (i.e., calves that are within 24 hours of birth). In addition, animals would be euthanized immediately if they exhibited signs of severe distress, including inability to stand, inability to access food or water, significant weight loss (>15%), labored breathing, or unrelieved pain. No animals reached these humane endpoints prior to the scheduled procedure. Newborn calves were euthanized using electrical stunning followed by exsanguination, in accordance with established animal welfare guidelines. Electrical stunning was applied to the head to induce immediate unconsciousness, as confirmed by the absence of corneal reflex and rhythmic breathing. The parameters of the electrical current (voltage, current intensity, and duration) were set according to standard veterinary recommendations to ensure rapid and humane euthanasia. To minimize suffering, animals were handled gently and procedures were performed by trained personnel. All experimental protocols were approved by the Institutional Animal Care and Use Committee (IACUC) and complied with relevant guidelines.

### 2.2. BMSCs culture and differentiation

The muscle tissue was placed in PBS containing 4% penicillin-streptomycin (Gibco, Thermo, USA) and quickly transferred into a UV-sterilized biosafety cabinet. The muscle tissue was minced in PBS containing 2% penicillin-streptomycin and the fascia and blood vessels were removed. The minced muscle tissue was then digested with 0.1% Type II collagenase (Solarbio, Beijing, China) diluted in DMEM/F12 basal medium (Gibco, Thermo, USA) at a tissue-to-digestive enzyme ratio of 1:5. The digestion process was carried out in a 37°C water bath for 30 min, with gentle shaking every 5 min. The digestion was stopped once no obvious tissue clumps remained. After the digestion was completed, DMEM/F12 containing 10% fetal bovine serum (Gibco, Thermo, USA) was added to terminate the digestion process. The resulting cell suspension was then filtered through a 70 μm filter and the filtered cell suspension was seeded into a T25 cell culture flask for incubation in a 37°C, 5% CO_2_ cell culture incubator. The growth medium was replaced every 2 d. When the cell density reached 70%−80%, passaging was performed. The cells were then passaged to the second generation and cryopreserved in liquid nitrogen for future use. Furthermore, when the second-generation BMSCs reached approximately 85% confluence, the growth medium was replaced with F12 differentiation medium containing 2% horse serum (Solarbio, Beijing, China) and cultured for 5 d. The differentiation status of muscle cells was monitored daily under a microscope.

### 2.3. Plasmid construction and cell transfection

The LNC297 overexpression plasmid (pc-LNC297), short interfering RNAs of GAS7 (si-GAS7), and mimic and inhibitor of miR-145 were synthesized by Beijing Qingke Biotechnology Co., Ltd. In the differentiation experiment, the second-generation BMSCs were cultured in a 6-well plate. When the cell density exceeded 80%, mimic, inhibitor, mimic NC, inhibitor NC, pc-LNC297, and siRNA were transfected into BMSCs according to the instructions of Lipofectamine 3000 (Invitrogen, Carlsbad, CA, USA). After 6 hours of transfection, the medium was replaced with differentiation medium containing 2% horse serum for 3 d of culture. The sequence information is shown in S1 Table.

### 2.4. Target gene prediction and luciferase reporter gene assay

This study used a dual-luciferase reporter system to explorethe interactions between miR-145 and GAS7, as well as between GAS7 and LNC297. The miRanda and Targetscan (http://www.targetscan.org/vert_71/) databases were employed to predict the target genes of miR-145 and their binding to the 3’ UTR of these target genes. Potential binding sites between miR-145 and LNC297 were predicted using DNASTAR and Lasergene v7.1 (based on sequence information). The luciferase reporter plasmids (wild type (WT) and mutant of the target sequence) were constructed by Qingke Biotechnology Co., Ltd. (Beijing, China). Next, 293T cells were seeded in a 24-well cell culture plate (NEST Biotechnology, Wuxi, China). When the cell density reached approximately 70%, miR-145mimic or negative control was co-transfected with specific WT or mutant plasmids according to the Lipofectamine 3000 reagent manual. After 48 h, luciferase activity was measured using the Duo-Lite TM luciferase detection system from Vazyme (Nanjing, China).

### 2.5. Real-time fluorescence quantitative analysis

Total RNA from the cells was extracted according to the manufacturer’s instructions of the RNAiso reagent kit (Takara, Beijing, China). The purity of the total RNA was assessed by measuring the A260/A280 and A260/A230 ratios using a NanoDrop 2000 UV-Vis spectrophotometer (Thermo, MA, USA). In addition, the integrity of the total RNA was assessed by 1.0% agarose gel electrophoresis.The total RNA and miRNA were reverse transcribed into first-strand cDNA using the PrimeScript RT reagent kit (Takara, Beijing, China) and the SYBR® PrimeScript™ miRNA RT-PCR Kit (Takara, Beijing, China), respectively, according to the manufacturer’s instructions. The qPCR analysis was performed using the CFX96 system (Bio-Rad, Hercules, CA, USA). The β-actin and U6 were used as reference genes for qPCR, with a reaction volume of 10 μL. The relative expression levels of each gene were analyzed using the 2^-ΔΔCt^ method. The mRQ 3’ primer from the Mir-X™ miRNA First-Strand Synthesis Kit (Takara, Beijing, China) was used as the reverse primer for miRNA measurement, and the primer information is shown in S2 Table.

### 2.6. Immunofluorescence

The BMSCs were seeded into 24-well cell culture plates. When the cell density reached approximately 60%−70%, they were removed. After washing three times with PBS, 4% cell fixative (Servicebio, Wuhan, China) was added to fix the cells for 15 min. After fixation, the cells were washed three times with PBS, followed by the addition of 0.2% permeabilization solution (Servicebio, Wuhan, China) for 10 min. Afterward, the cells were washed three times with PBS, each time for 5 min. Add blocking solution (Servicebio, Wuhan, China) and incubate on a shaker for1 h. Then wash with PBS three times, each time for 5 min. Add 200 μL of the primary antibody diluted with antibody dilution buffer (Servicebio, Wuhan, China) to each well and incubate overnight at 4°C on a shaker. Afterward, add the fluorescent secondary antibody [FITC-conjugated Goat anti-Rabbit IgG (H + L)] and incubate for 2 h at room temperature in the dark. Then, add 250 μL of DAPI (Beyotime, Shanghai, China) for staining for 15 min. Finally, wash with PBS twice, each time for 5 min, then add a small amount of PBS and place the cell culture plate under an inverted fluorescence microscope to capture images of green fluorescence (positive cells, excitation wavelength = 488 nm) and blue fluorescence (nucleus, excitation wavelength = 358 nm) in the same field of view.

### 2.7. RNA pull-down

The RNA pull-down Kit (GeneCreate, Wuhan, China) was used to verify the targeting relationship between miR-145 and LNC297. The biotin-labeled mimic of miR-145 was synthesized by Qingke Biotechnology Co., Ltd. The BMSCs were seeded into 10 cm culture dishes. When the cell density reached 60%, transfection was performed using Lipofectamine 3000 according to the manufacturer’s instructions. In the experimental group, the biotin-labeled miRNA probe and pc-LNC297 were co-transfected, while the control group was transfected with mimic NC and empty plasmid. After 48 h of culture, the cells were collected by trypsin digestion and washed twice with precooled PBS. The following steps were performed according to the manufacturer’s instructions for the RNA pull-down Kit. In brief, the collected cells were lysed, magnetic beads were incubated with the probes, RNA was extracted, the first strand of cDNA was synthesized, and qPCR was performed to verify whether miR-145 binds to LNC297.

### 2.8. Western blot analysis

Total protein from cells was extracted using a total protein extraction kit (Solarbio, Beijing, China), and the protein concentration was measured using a protein assay kit (Bestbio, Shanghai, China). Only samples with protein concentrations greater than 10 mg/mL were used for subsequent experiments. In brief, the protein samples were prepared by mixing total protein with SDS-PAGE loading buffer (Biosharp, Shanghai, China) in 4:1 ratio, and then the samples were denatured at 99°C for 10 min. Denatured proteins were separated using a10% one-step gel electrophoresis prep kit (ORISCIENCE, Chengdu, China). The separated gel was used for protein transfer to a 0.22 μm PVDF membrane via a wet transfer method (electrophoresis conditions: 400 mA, 60 min). The membrane was gently incubated at room temperature for 1.5 hours in Tris-buffered saline with Tween (TBST) containing 5.0% skimmed milk (Solarbio, Shanghai, China). The membrane was then incubated overnight at 4°C with the corresponding primary antibody on a shaking platform. After primary antibody incubation, the membrane was washed three times with TBST for 5 min each. The membrane was then incubated for 1.5 h with the secondary antibody [Goat anti-Rabbit or anti-Mouse IgG H&L (HRP), Zen Bioscience, Chengdu, China]. Finally, the membrane was washed three times with TBST. Immunoreactions were detected using ECL chemiluminescence reagent (HAKATA, Shanghai, China), and images were captured using the Touch Imager Pro device from e-BLOT Biotech (Shanghai, China).WB grayscale values were statistically calculated using Image J (v 1.53, Bethesda, MD, USA). Specific information about the primary antibody is provided in S3 Table.

### 2.9. Cell culture, BHBA and NAHB treatment and transfection

BMSCs were cultured in growth medium containing 10% FBS in a cell incubator at 37°C with 5% CO_2_. For BHBA (773861, Sigma, USA) treatment, when the BMSCs reached 85–90% confluence, differentiation induction medium containing 2% horse serum was added and cultured for 60 h. The differentiation medium was then replaced with medium containing 0 mM,1.2 mM, 2.4 mM, or 4.8 mM BHBA and cultured for 12 h. For NAHB (54965, Sigma, USA) treatment, after 60 h of differentiation, the differentiation medium was replaced with medium containing 4.8 mM NAHB and cultured for 12 h. To investigate the co-treatment of LNC297, miR-145, and GAS7 with BHBA, BMSCs were transfected and induced to differentiate as described in method 2.2, and after 60 h of differentiation, the differentiation medium was replaced with induction differentiation medium containing 4.8 mM BHBA and cultured for an additional 12 h. BHBA and NAHB were used to treat BMSCs for 12 h to investigate their effects on differentiation. This was based on previous studies and the metabolic time of BHBA in the body [26].

### 2.10. Statistical analysis

Statistical analysis was performed using GraphPad (v10.4.0) software, with intergroup differences assessed by Student’s t-test. In addition, multiple comparisons were performed using one-way or two-way ANOVA analysis. When * *P* < 0.05, the differences were significant. All data are presented as means ± SEM.

## 3. Results

### 3.1. BHBA inhibits the differentiation of BMSCs

**Fig 1:**
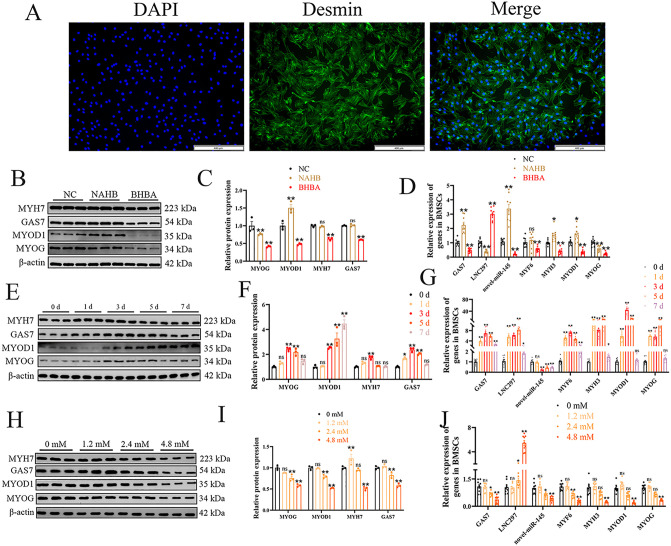
BHBA inhibits the differentiation of BMSCs. (A) Immunofluorescence staining of BMSCs. microscopy images showing the localization of Desmin (green) in BMSCs. The nuclei were counterstained with DAPI (blue) (100×). (B, C) On the third day of BMSCs differentiation, 12 h after treatment with 4.8 mM BHBA and NAHB, Western blot analysis and gray value detection were conducted (n = 3). (D) The relative expression levels of *GSA7*, miR-145, LNC297 and myogenic differentiation-related genes (n = 9). (E, F) Western blot analysis and gray value detection of differentiation marker genes on days 0, 1, 3, 5, and 7 of BMSCs differentiation, as well as GAS7 (n = 3). (G) The relative expression levels of differentiation marker genes on days 0,1, 3, 5, and 7 of BMSCs differentiation, as well as the genes *GAS7*, LNC297, and miR-145 (n = 9). (H, I) Western blot analysis and gray value detection were conducted (n = 3). The data presented as means ± SEM. * *P* < 0.05; ** *P* < 0.01.

The BMSCs in this study were obtained from the hind limb skeletal muscle of neonatal Chinese Holstein cows. To verify their identity as BMSCs, Desmin muscle-specific antibody was used. Desmin is a unique intermediate filament protein in muscle cells, widely present in skeletal muscle, cardiac muscle, and smooth muscle cells [27]. The results show that the cells used in this study are BMSCs (Fig 1A). Previous studies have indicated that both the acidic BHBA and the neutral sodium salt (NAHB) might be used in in vitro experiments with 3-hydroxybutyrate [28,29]. To investigate which substance better reflects the effect of 3-hydroxybutyrate on muscle differentiation in a ketosis cow model, BMSCs were treated with 4.8 mM BHBA and NAHB for 12 h, respectively. The Western blot results revealed that BHBA significantly inhibited the expression of MYOG, MYOD1, MYH7, and GAS7, while NAHB, except for inhibiting the expression of MYOG, had no significant effect on the protein expression of other genes (Fig 1B-D). The qPCR results show that BHBA inhibits the differentiation of BMSCs, promotes the expression of LNC297, and inhibits the expression of *GAS7*, while NAHB shows the opposite results. Therefore, this result suggests that BHBA is more suitable for further progressed, the expression of differentiation marker genes first increased and then decreased, with the best differentiation observed on day 3. The qPCR results indicated that *GAS7* and LNC297 were positively correlated with BMSCs differentiation, while miR-145 was negatively correlated with differentiation (Fig 1E-G). To further explore the effect of BHBA on BMSCs differentiation, BHBA concentrations of 0 mM, 1.2 mM, 2.4 mM, and 4.8 mM were applied to BMSCs for 12 h on the third day of differentiation. The results showed that the higher the concentration, the more significant the inhibitory effect of BHBA on BMSCs differentiation. Additionally, LNC297, *GAS7,* andmiR-145 also exhibited dose-dependent effects (Fig 1H-J). The above results suggest that BHBA inhibits BMSCs differentiation in a dose-dependent manner, and GAS7, LNC297, and miR-145 might influence BMSCs differentiation.

### 3.2. miR-145 enhances the inhibitory effect of BHBA on BMSCs differentiation

**Fig 2:**
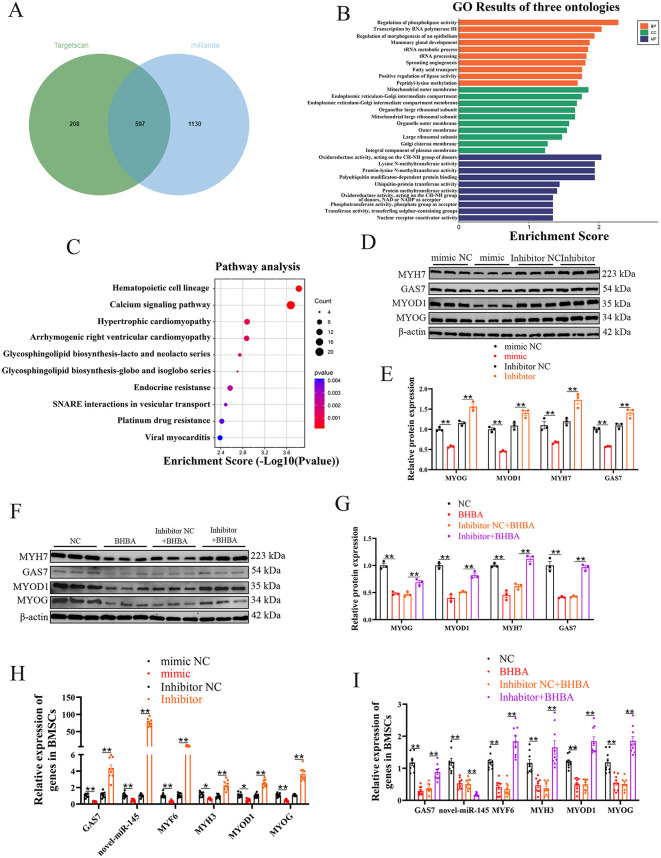
miR-145 enhances the inhibitory effect of BHBA on BMSCs differentiation. (A) Venn diagram of the number of target gene predictions from the Targetscan and miRanda databases. (B) GO analysis. (C) KEGG analysis of the differentially expressed genes only listed significant enrichment pathways. (D, E) After overexpressing and inhibiting the expression of miR-145, WB analysis of GAS7 and differentiation-related genes, as well as grayscale value detection (n = 3). (F, G) After inhibiting the expression of miR-145, and co-treating with BHBA for 12 h, WB analysis of GAS7 and differentiation-related genes and grayscale value detection (n = 3). (H, I) The relative expression levels of *GSA7*, miR-145 and myogenic differentiation-related genes (n = 9). The data presented as means ± SEM. * *P* < 0.05; ** *P* < 0.01.

To further explore the function of miR-145, the downstream target genes were predicted using the Targetscan and miRanda databases. A total of 597 target genes were predicted in both databases (Fig 2A). GO and KEGG analyses were performed on these 597 target genes. These target genes are located in the mitochondrial outer membrane, endoplasmic reticulum-Golgi intermediate compartment, organelle outer membrane, and other regions. They are involved in biological processes such as tRNA metabolic process, regulation of phospholipase activity, transcription by RNA polymerase III, ncRNA processing, and others. They possess molecular functions like oxidoreductase activity, acting on the CH-NH group of donors, lysine N-methyltransferase activity, and protein-lysine N-methyltransferase activity. KEGG results showed that these genes are significantly enriched in pathways such as Calcium signaling pathway, Hypertrophic cardiomyopathy, and Viral myocarditis (Fig 2B, C). The differentiation of muscle cells is an essential process for muscle tissue generation, ensuring the structural integrity, functional characteristics, and repair ability of muscles. To explore the role of miR-145 in BMSCs differentiation, we transfected the mimic, mimic NC, Inhibitor, and Inhibitor NC of miR-145 into BMSCs and then cultured them in differentiation medium for 3 days. The results showed that the expression of miR-145 was significantly upregulated in the mimic group and significantly downregulated in the Inhibitor group, indicating successful transfection. Furthermore, when the expression of miR-145 was increased, the expression of muscle differentiation marker genes decreased, while inhibiting its expression showed the opposite trend. Additionally, the expression of GAS7 was inversely correlated with the expression of miR-145, further suggesting that GAS7 might be a downstream target gene of miR-145 (Fig 2D, E, H). To further verify whether miR-145 could inhibit the effect of BHBA on muscle cell differentiation, we added 4.8 mM BHBA to the Inhibitor group and co-treated for 12 hours. The results showed that when the expression of miR-145 was inhibited, the inhibitory effect of BHBA on muscle cell differentiation was relieved (Fig 2F, G, I). These results suggest that miR-145 inhibits BMSCs differentiation and enhances the inhibitory effect of BHBA on BMSCs differentiation. Moreover, *GAS7* may be a downstream target gene of miR-145.

### 3.3. LNC297 overexpression alleviates the inhibitory effect of BHBA on BMSCs differentiation, while GAS7 knockdown exacerbates this inhibition

**Fig 3:**
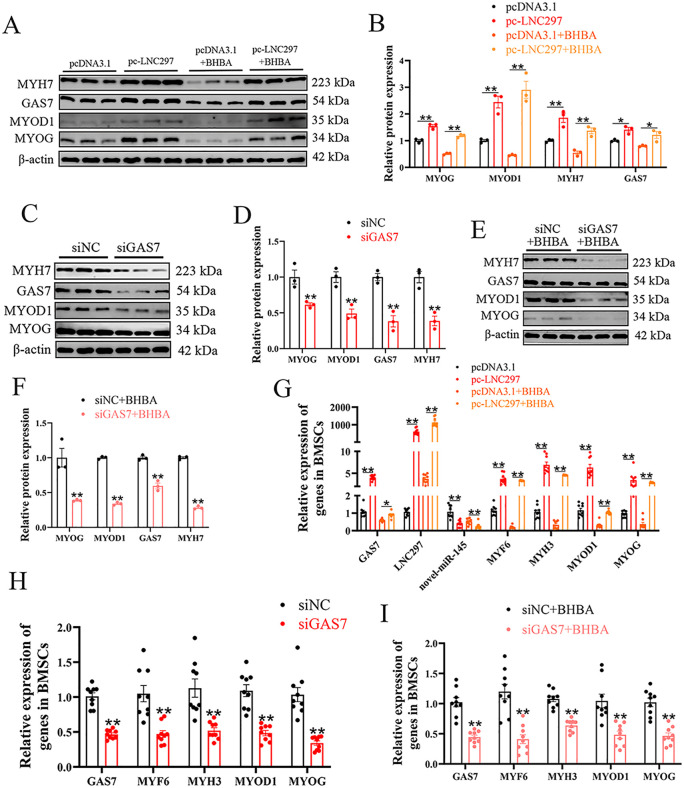
LNC297 and GAS7 alleviate the inhibitory effect of BHBA on the differentiation of BMSCs. (A-F) WB analysis of GAS7 and differentiation-related genes, as well as grayscale value detection (n = 3). (G-I) The relative expression levels of *GSA7*, miR-145 and myogenic differentiation-related genes (n = 9). The data presented as means ± SEM. * *P* < 0.05; ** *P* < 0.01.

To explore the role of LNC297 in the differentiation of BMSCs, after overexpressing LNC297, the expression of differentiation marker genes was increased, indicating that LNC297 promotes BMSCs differentiation. Furthermore, BHBA inhibited this differentiation, but after co-treatment with LNC297 overexpression and BHBA, differentiation-related genes were significantly upregulated, further suggesting that LNC297 can regulate the myogenic differentiation process (Fig 3A, B). The expression of GAS7 increased during differentiation. To further investigate its impact on BMSCs differentiation, we used interfering RNA to reduce the expression of GAS7. WB results showed successful inhibition of GAS7 expression, while the expression of differentiation-related genes decreased, and differentiation was suppressed. After co-treatment with BHBA, the differentiation of BMSCs was further inhibited (Fig 3C-F). The qPCR results were consistent with the WB results. Additionally, after overexpressing LNC297, the expression level of miR-145 was downregulated (Fig 3G-I). These results suggest that LNC297 and GAS7 promote BMSCs differentiation and attenuate BHBA-induced inhibition, which might help alleviate muscle tissue damage in bovine ketosis. Moreover, LNC297 may regulate the expression of miR-145 to exert its effect.

### 3.4. LNC297 can promote the differentiation of BMSCs through the miR-145/GAS7 pathway

**Fig 4:**
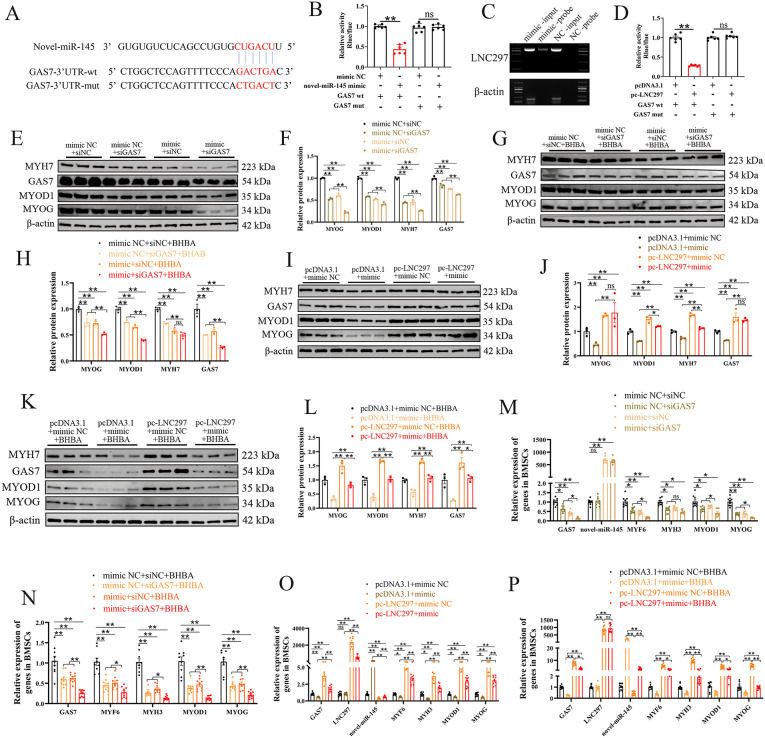
LNC297 promotes the differentiation of BMSCs through the miR-145/GAS7 pathway. (A) The predicted binding site of gene GAS7 with miR-145. (B) Luciferase assay was conducted by co-transfecting GAS7 WT and mutant plasmids with miR-145 mimic and NC into 293T cells, with the WT + NC group serving as the control (n = 6). (C) The miRNA pull-down experiment results show that LNC297 binds to miR-145. Biotin-labeled miR-145 or control miRNA (mimic NC) was transfected into BMSCs, followed by affinity capture and PCR detection of LNC297 expression. (D) Luciferase assay was conducted by co-transfecting GAS7 WT and mutant plasmids with pc-LNC297 and pcDNA3.1 into 293T cells, with the WT + NC group serving as the control (n = 6). (E-L) WB analysis of GAS7 and differentiation-related genes, as well as grayscale value detection (n = 3). (M-P) The relative expression levels of *GSA7*, miR-145 and myogenic differentiation-related genes (n = 9). The data presented as means ± SEM. * *P* < 0.05; ** *P* < 0.01.

The dual luciferase reporter assay confirmed the targeting relationship between miR-145 and GAS7 (Fig 4A, B). The results of the miRNA pull-down experiment show that LNC297 targets and binds with miR-145 (Fig 4C). Additionally, the dual luciferase reporter assay indicates that LNC297 has a targeting relationship with GAS7 (Fig 4D). To further verify the regulatory effect of miR-145 on GAS7, co-transfection experiments with mimic and siGAS7 were performed. Western blot results show that when mimic and siGAS7 were co-transfected, the expression levels of MYOG, MYOD1, MYH7, and GAS7 were the lowest, suggesting that miR-145 targets and inhibits the expression of GAS7, thereby suppressing the differentiation of BMSCs (Fig 4E, F). Similarly, the results of co-transfection of mimic and siGAS7 followed by 12-hour BHBA treatment were consistent with the above results (Fig 4G, H). To verify the mechanism of action of LNC297 and miR-145, this study co-transfected overexpressed LNC297 (pc-LNC297) and the overexpression mimic of miR-145 into BMSCs. The results showed that LNC297 could alleviate the inhibitory effect of miR-145 on the differentiation of BMSCs. Additionally, LNC297 inhibited the expression of miR-145 and promoted the expression of GAS7 (Fig 4I-P). In conclusion, LNC297 can target and competitively bind to miR-145 to promote the expression of GAS7, thereby promoting the differentiation of BMSCs and alleviating the inhibition of muscle differentiation under BHBA stress.

## 4. Discussion

During late gestation and early postpartum, dairy cows frequently experience negative energy balance (NEB) as energy demands exceed intake, leading to mobilization of body fat reserves [30]. However, fat mobilization alone cannot adequately support the combined energy and nutrient requirements of fetal growth, mammary gland development, and early lactation. The previous studies have shown that during negative energy balance (NEB), the muscle tissue weight of dairy cow’s decreases, indicating muscle atrophy. This is primarily because cows with ketosis break down muscle tissue to provide the necessary amino acids and other substances [31,32]. Skeletal muscle mobilization in transition dairy cows is relatively limited, and most studies have been conducted within feeding trials. In-depth investigations into the underlying mechanisms are still lacking [33]. BHBA is a ketone body generated in the liver during fat mobilization in cows experiencing NEB. Elevated blood BHBA concentrations are therefore a key indicator of ketosis.

However, BHBA might serve as an energy substrate for oxidation in muscle tissue, but the specific mechanisms and whether it affects muscle formation are still unclear. This study uses BHBA as an experimental treatment, in both its acidic and sodium salt forms, to model BHBA accumulation in muscle under in vitro conditions. However, previous studies have shown that NAHB, as a metabolic substrate, might enhance the energy efficiency of myogenic cells by stimulating the expression of G protein-coupled receptor 109a (GPR109a), thereby upregulating 3-HB transporters MCT1 and CD147, utilizing the enzyme OXCT1 and phosphorylated AMPK to increase ATP production. As a signaling molecule, NAHB activates GPR109a, promoting calcium influx, improving calcium homeostasis, and increasing the expression of calcium-related proteins such as CAMKK2. This signaling process activates calcium-calmodulin-dependent phosphatase (CaN), promoting the translocation of NFAT to the cell nucleus and gene expression, thereby stimulating the proliferation and differentiation of myogenic cells [26]. However, the reduced expression of MYH7 and MYH4 in the muscle tissue of Cows with ketosis indicates a decline in muscle contraction ability and potential damage such as muscle atrophy or degeneration. Myocyte differentiation is a key biological process for repairing muscle damage; therefore, it is speculated that the differentiation of skeletal muscle in cows with ketosis is suppressed, while NAHB treatment promotes this process. Thus, it is inferred that NAHB does not align with the metabolic pattern of β-hydroxybutyrate in the muscle tissue of Cows with ketosis [8]. The results of this study also confirm that NAHB promotes myocyte differentiation, consistent with previous BHBA treatment inhibits muscle differentiation, which aligns with the findings from our previous studies. Therefore, BHBA is more suitable for exploring the metabolic pattern of 3-hydroxybutyricacid in the muscle tissue of cows with ketosis. Additionally, the low concentrations of BHBA (1.2 mM-2.4 mM) have little effect on the differentiation of BMSCs, possibly because low concentrations of BHBA are important metabolic products of ketone bodies, serving as an energy source for cells and providing ATP to support normal cellular function. Especially under conditions of glucose scarcity (such as during fasting or intense exercise), ketone bodies act as an alternative energy source, maintaining the cell’s energy balance and thus not interfering with normal cellular differentiation processes. However, high concentrations of BHBA, especially when cows are affected by ketosis, may lead to metabolic acidosis, which is the accumulation of acidic substances in the blood. This acidosis inhibits the normal function of skeletal muscle satellite cells, hindering their differentiation. This metabolic stress causes damage to cells. In our experimental design, the 12-hour treatment was intended as a preliminary induction period to assess early cellular responses within a short timeframe, and to inform future experiments involving longer treatment durations.

Muscle tissue constitutes 40% of bodyweight in mammals and plays a vital role in regulating metabolism and maintaining internal balance [34]. The formation of skeletal muscle begins with the determination of muscle cell fate, a process regulated by the myogenic transcription factors Pax7 and MYOD1. Subsequently, the expression of numerous genes establishes the structure and function of the muscle [35,36]. A key step in this process is the fusion of mononucleated muscle cells to form multinucleated muscle fibers. This study found that as BMSCs differentiate, the differentiation effect of MYH7, MYOD1, and MYOG is most prominent on day 3. Therefore, day 3 of differentiation was chosen as the time point to investigate the mechanism of BHBA’s effect on differentiation.

In addition, when damaged, muscle-derived progenitor cells in adult muscle tissue are activated and fuse to generate new muscle fibers [37]. Therefore, BHBA will affect the differentiation of muscle tissue in ketosis-affected cows. Therefore, BHBA may affect the differentiation of muscle tissue in dairy cows with ketosis, thereby potentially impairing muscle function to some extent. However, we speculate that the muscle repair mechanism might be activated following BHBA-induced stress. Upon muscle injury, the activation and proliferation of satellite cells are critical steps in muscle regeneration. Nevertheless, this study has not yet explored the effect of BHBA on the activity of BMSCs and their role in muscle repair. Therefore, future studies are needed to further clarify the impact of BHBA on BMSC activity and their potential involvement in the muscle regeneration process, in order to improve our understanding of the role of BHBA in muscle repair mechanisms. The proliferating satellite cells enter the cell cycle, differentiate into myoblasts, and subsequently fuse to form new muscle fibers, aiding in the repair of the damaged muscle tissue [38]. Most mammalian genomes transcribe a large amount of non-coding RNA (ncRNA), such as microRNA, circular RNA, and long non-coding RNA. Among these, long non-coding RNA is the most functionally diverse category and plays an important role in the regulation of development, differentiation, and maintenance of cell specificity [39]. For example, Lnc-MEG8 targets and binds to miR-22-3p, thereby reducing the inhibitory effects of miR-22-3p on the key myogenic factors MYF5 and MYOG, which promotes myotube formation [40]. Through previous studies, we identified an unannotated long non-coding RNA, LNC297, whose expression level increases during differentiation. Additionally, after BHBA treatment, the expression of LNC297 rises with the increasing concentration of BHBA, indicating its important role in the differentiation process of BMSCs and its potential involvement in regulating muscle formation in ketosis-affected cows. Furthermore, we found that overexpression of LNC297 significantly upregulates the expression levels of muscle differentiation marker genes such as MYOG, MYOD1, and MYH7. After combined treatment with BHBA, the expression of muscle differentiation-related genes still showed an upward trend, suggesting that LNC297 can promote BMSCs differentiation and partially alleviate the inhibitory effects of BHBA on muscle differentiation.

This study found that miR-145 significantly inhibits the differentiation ability of BMSCs, and under conditions of co-treatment with BHBA, the differentiation of BMSCs is further inhibited, indicating that the two may have a synergistic inhibitory effect in regulating muscle differentiation. Existing research has shown that miR-145 plays an important role as a negative regulatory factor in various cell types, particularly in myogenesis, where miR-145 interferes with the process of cellular differentiation into muscle by targeting muscle-specific genes or affecting the expression of Myogenic Regulatory Factors (such as MYOD, MYOG) [41,42]. The miR-145 newly discovered in this study belongs to the miR-145 family and is likely involved in regulating important biological processes such as cell proliferation, differentiation, and apoptosis through similar mechanisms. In particular, both miR-145 and miR-145 may play analogous roles in the regulation of muscle cell differentiation, cancer biology, and the proliferation of vascular smooth muscle cells. In this study, it was found that the expression of miR-145 is downregulated as differentiation progresses, and it also shows a downward trend under BHBA treatment. Therefore, it is initially speculated that miR-145 inhibits BMSCs differentiation. The results from Western blotting and qPCR also confirmed that miR-145 suppresses the expression of muscle differentiation marker genes, thereby inhibiting BMSCs differentiation. Furthermore, after co-treatment with BHBA, the expression of muscle differentiation-related genes further showed a downregulation trend, suggesting that miR-145 might promote BMSCs differentiation and exacerbate the inhibitory effects of BHBA on muscle differentiation.

Some studies have reported that BHBA can promote cell antioxidant capacity and survival under certain conditions, under the conditions of this study, BHBA, whether administered alone or in combination with miR-145, exhibited an inhibitory effect on BMSCs differentiation. This suggests that BHBA might amplify the inhibitory effects mediated by miR-145 by affecting metabolic pathways and interfering with muscle cell differentiation signals (such as mTOR, AMPK, Wnt). Notably, BHBA may regulate the expression or stability of miRNAs, or influence the efficiency of miRNA target gene expression by altering the intracellular metabolic state, thereby enhancing the functionality of miR-145. This observation indicates a potential complementary or intersecting relationship between the two, providing new clues for understanding the coordinated regulation of muscle cell fate by miRNAs and metabolites. However, the negative impact of miR-145 on the differentiation process does not necessarily equate to a reduction in cell viability. It may exert a protective effect on undifferentiated cells, where the enhanced antioxidant capacity could negatively influence differentiation while simultaneously promoting cell survival. The relative proportion between undifferentiated and differentiated cells may significantly affect the outcomes of the current study. Therefore, the lack of in-depth investigation into the effects of miR-145 on BMSCs proliferation and apoptosis, as well as its role in the muscle damage and repair mechanisms in dairy cows with ketosis, represents a limitation of this study and warrants further investigation.

miRNA exerts its functions through various mechanisms. Typically, miRNAs inhibit gene expression by binding to the 3’ UTR of target mRNAs. This interaction involves specific complementary pairing between the miRNA and its target mRNA, often centered around a particular “seed sequence.” Such binding can lead to the repression of mRNA translation or its degradation, ultimately influencing the protein products and thereby regulating biological processes including cell differentiation, apoptosis, and proliferation [43]. In this study, the online prediction tools TargetScan and miRanda were utilized to identify the target genes of miR-145. Subsequently, GO and KEGG analyses were conducted to perform functional enrichment analysis of these target genes, allowing us to infer the potential functions of miR-145. The results indicated that these target genes were significantly enriched in pathways such as hypertrophic cardiomyopathy, arrhythmogenic right ventricular cardiomyopathy, calcium signaling pathway, and viral myocarditis. These pathways have been recognized as being critical for muscle cell differentiation [44,45]. Additionally, this study identified a key target gene, *GAS7*, which is involved in the PI3K/AKT signaling pathway, a pathway closely associated with muscle development [46,47]. The results of this study indicate that the overexpression of miR-145 leads to a decrease in both the mRNA and protein levels of *GAS7*. Furthermore, the dual-luciferase assay revealed that there is a targeted relationship between miR-145 and *GAS7*. Additionally, Western blot and quantitative results demonstrated that *GAS7* promotes the differentiation of BMSCs. Therefore, based on these findings, we conclude that miR-145 exerts an inhibitory effect on the differentiation of BMSCs by directly targeting *GAS7*.

In recent years, an increasing body of evidence has shown that long non-coding RNAs (lncRNAs), as ceRNAs interacting with microRNAs (miRNAs), play a significant role in the development and differentiation of muscle and fat by regulating the expression of their target mRNAs [48]. The interaction between lncRNA, miRNA, and mRNA plays acritical role in various biological processes during development and disease. It has been recognized as a common regulatory mechanism in the functional control of lncRNAs [49,50]. The results of this study indicate that LNC297 binds to miR-145 and inhibits its expression, leading to the upregulation of the GAS7 gene, thereby regulating the differentiation of BMSCs. This study also explored the targeted relationship between LNC297 and GAS7; however, whether LNC297 directly regulates GAS7 under BHBA stress to further alleviate the inhibitory effects on muscle differentiation still requires further experiments for validation.

## 5. Conclusion

In summary, this study established that BHBA can inhibit the differentiation of BMSCs and developed an in vitro BHBA stress model, providing a theoretical basis for exploring the developmental mechanisms of muscle tissue in Cows with ketosis. Additionally, it identified LNC297 as a key factor in BMSCs differentiation that can alleviate the impairment of muscle differentiation under high BHBA stress. This research is the first to investigate the overexpression of LNC297 in BMSCs to promote differentiation, effectively enhancing the muscle differentiation process when co-treated with BHBA. Furthermore, it revealed a novel interaction between LNC297 and miR-145. We provided evidence that LNC297 regulates the expression of GAS7 in BMSCs by acting as a sponge for miR-145, thereby exerting its regulatory effects. This study enriches the understanding of long non-coding RNAs and competitive endogenous RNAs in the development of BMSCs and offers new targets for the treatment of Cows with ketosis and other metabolic syndromes.

## Supporting information

S1 Table Sequence information for plasmids, siRNAs, miRNA mimics, inhibitors, and negative controls used in this study. (XLSX)

S2 Table Primer sequences used for real-time quantitative PCR analysis. (XLSX)

S3 Table Information on primary antibodies used for Western blot analysis and immunofluorescence staining. (XLSX)

S1 Fig Original uncropped and unadjusted blot and gel images underlying the Western blot and gel results reported in this study. (PDF)

## Abbreviations

BHBA: β-hydroxybutyrate
NAHB: Sodium β-hy297
BMSCs: Bovine skeletal muscle satellite cells
NEB: Negative energy balance
LNC297: LncRNA MSTRG 29792.14
ceRNA: Competing endogenous RNA
lncRNAs: Long non-coding RNAs
NEFA: Non-esterified fatty acids
