# Supplementary material for: LNC297 promotes BMSCs differentiation and alleviates BHBA-induced inhibition through the miR-145/GAS7 axis
Source: PLoS One. 2026 Jul 22;21(7):e0354035. doi: 10.1371/journal.pone.0354035 (PMC13390936; doi:10.1371/journal.pone.0354035)
Supplement: S1 Fig — (PDF) [file pone.0354035.s004.pdf]

Figure 1B

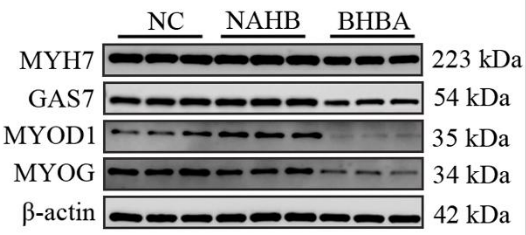

Figure in manuscript

Group	actin	MYOG	MYOD1	GAS7	MYH7
NC	133334	101391	64810	114185	181403
NC	123248	98882	65200	105898	178526
NC	124698	136945	77068	109508	180396
NAHB	127411	88618	100802	115511	180050
NAHB	133797	87203	99803	116894	185163
NAHB	133718	89818	123034	113283	180598
BHBA	119933	46203	31584	61804	99374
BHBA	125980	46484	30894	66669	116886
BHBA	121907	41981	33407	62724	113776

The original data of the gray values of each WB band in Figure 1B

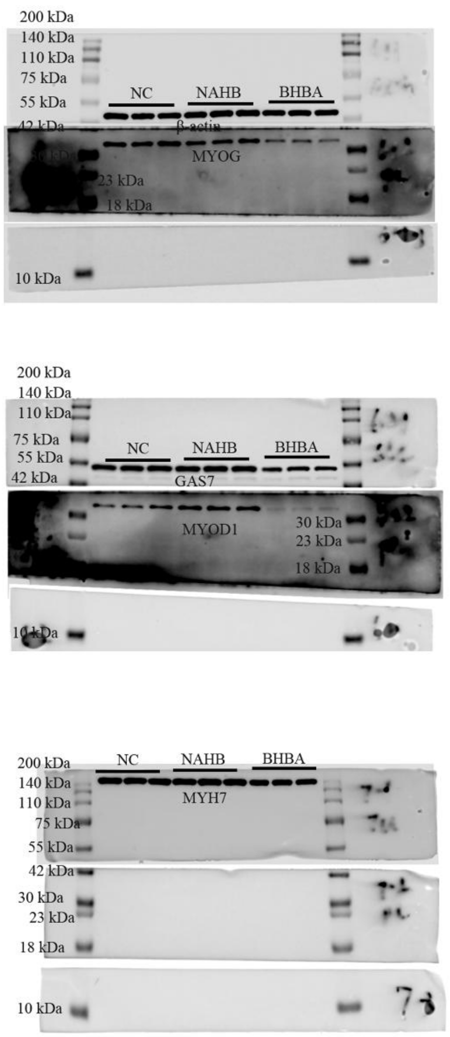

Original images

Figure 1E

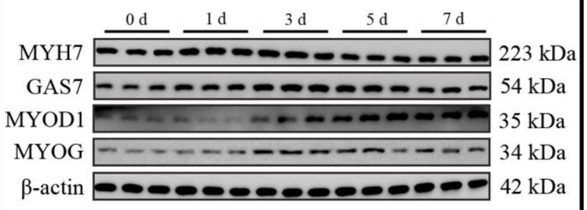

Figure in manuscript

Group	actin	MYOG	MYOD1	GAS7	MYH7
0d	115442	35323	35660	50096	75007
0d	113454	31473	35091	48741	70601
0d	113309	31263	43497	53868	77732
1d	118871	41627	41555	86919	95295
1d	115042	42339	39633	82569	99360
1d	106779	43615	37549	84321	99326
3d	108108	77200	88348	120149	131018
3d	109796	79634	94137	122169	131929
3d	116311	74974	100878	113258	120229
5d	115660	73341	104150	107487	79217
5d	113009	79855	110269	106091	78003
5d	113595	61595	158434	99477	80016
7d	114977	40082	156246	57781	75666
7d	114055	50688	162409	60691	71860
7d	103574	47708	175547	55310	72600

The original data of the gray values of each WB band in Figure 1E

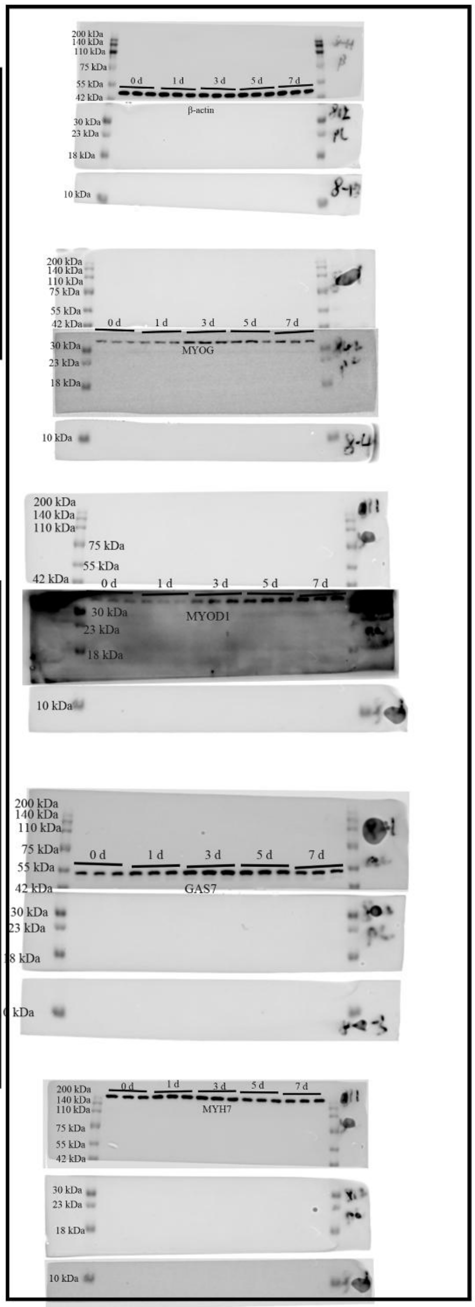

Original images

Figure 1H

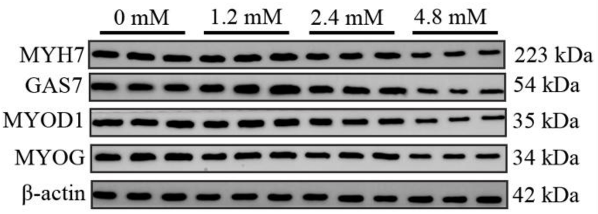

Figure in manuscript

Group	actin	MYOG	MYOD1	GAS7	MYH7
0 mM	493313	550889	597375	631807	545328
0 mM	488672	571872	550942	600541	584794
0 mM	484833	604472	598198	580006	556698
1.2 mM	448077	461509	520837	595374	518183
1.2 mM	479763	495464	572025	699741	562973
1.2 mM	468557	491734	548192	817215	587703
2.4 mM	464724	399495	461738	537453	437007
2.4 mM	496125	406020	443818	570355	431001
2.4 mM	465130	461701	439475	566718	479731
4.8 mM	477609	307390	302216	313489	316159
4.8 mM	446713	323690	270742	256560	324973
4.8 mM	499304	344073	310208	338058	309266

The original data of the gray values of each WB band in Figure 1H

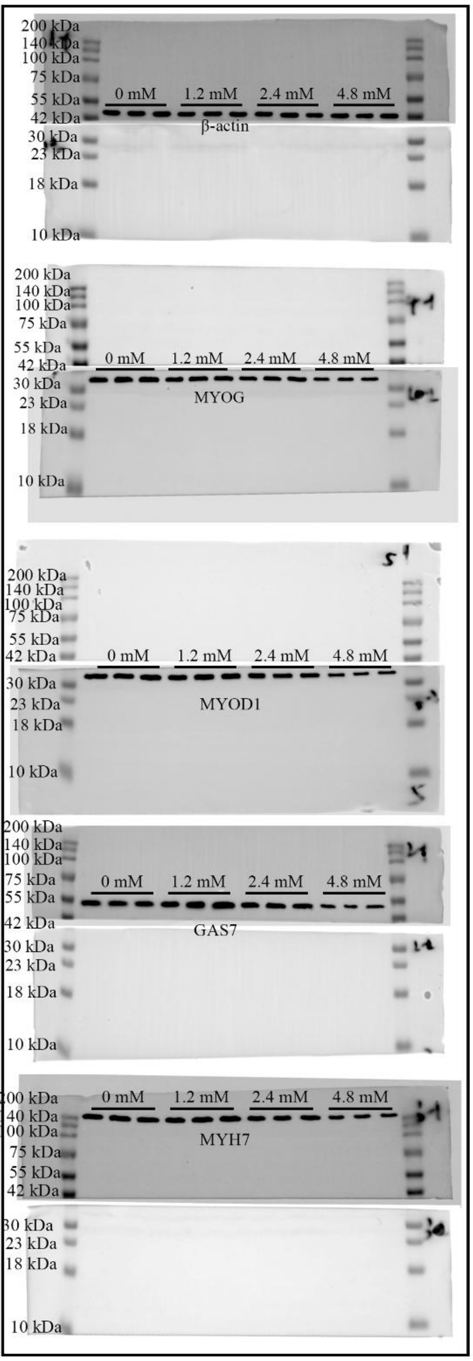

Original images

Figure 2D

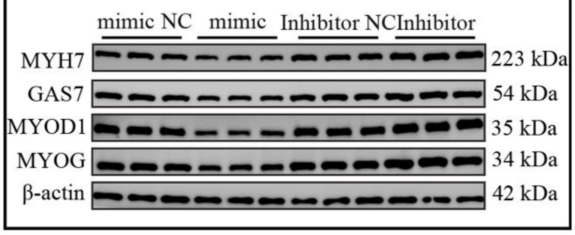

Figure in manuscript

Group	actin	MYOG	MYOD1	GAS7	MYH7
mimic NC	145753	171621	179144	139040	120446
mimic NC	139563	178837	163663	134670	123516
mimic NC	147903	172286	159339	128843	123506
mimic	135691	89145	69470	71071	73640
mimic	132669	92149	68856	71489	78502
mimic	133982	96720	74895	72925	77575
Inhibitor NC	121285	177380	166168	128336	144063
Inhibitor NC	128154	179461	163681	134805	139362
Inhibitor NC	140126	184155	160964	131351	142674
Inhibitor	140354	247608	209072	166203	182142
Inhibitor	123227	229048	209280	168998	187027
Inhibitor	124294	253342	210817	171591	197159

The original data of the gray values of each WB band in Figure 2D

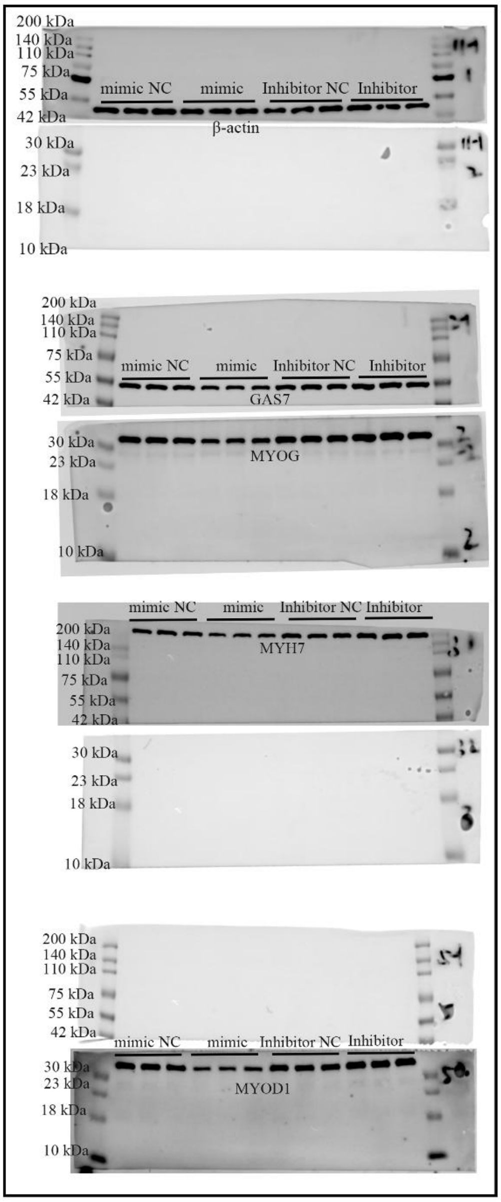

Original images

Figure 2F

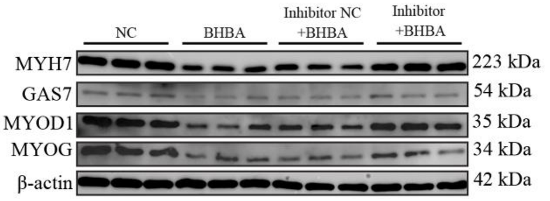

Figure in manuscript

Group	actin	MYOG	MYOD1	GAS7	MYH7
NC	242457	234191	267378	115731	229809
NC	238659	209974	249343	123668	229192
NC	227538	192559	223152	137892	232335
BHBA	231987	98902	79471	49687	82133
BHBA	221134	102367	91035	48378	116236
BHBA	221481	91276	108578	51288	101811
Inhibitor NC+BHBA	211071	86628	109997	47978	136651
Inhibitor NC+BHBA	199542	92925	110254	47347	124926
Inhibitor NC+BHBA	207162	79564	109460	45636	112592
Inhibitor+BHBA	215528	143974	176721	116623	216202
Inhibitor+BHBA	193798	122143	180276	102277	220627
Inhibitor+BHBA	208396	116293	168364	99959	238783

The original data of the gray values of each WB band in Figure 2F

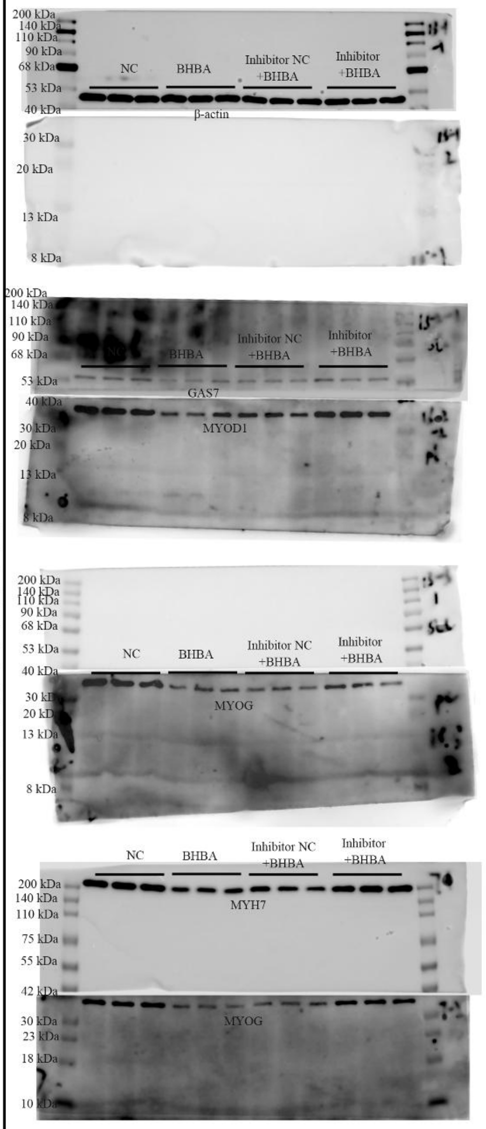

Original images

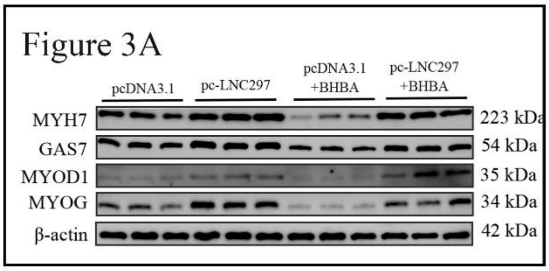

Figure in manuscript

Group	actin	MYOG	MYOD1	GAS7	MYH7
pcDNA3.1	161896	96624	53608	119709	115288
pcDNA3.1	173956	101687	51449	120106	121238
pcDNA3.1	163916	84679	56176	111585	104711
pc-LNC297	216887	174814	140308	190318	224185
pc-LNC297	174814	159091	149702	179880	235422
pc-LNC297	173064	157059	150288	185902	247139
pcDNA3.1+BHBA	169586	50742	24566	92543	48788
pcDNA3.1+BHBA	171715	45672	24211	96476	66735
pcDNA3.1+BHBA	153919	46233	24255	89954	68177
pc-LNC297+BHBA	152657	106239	133316	140289	158284
pc-LNC297+BHBA	144508	98211	165033	139980	141753
pc-LNC297+BHBA	199997	125698	160364	135032	158112

The original data of the gray values of each WB band in Figure 3A

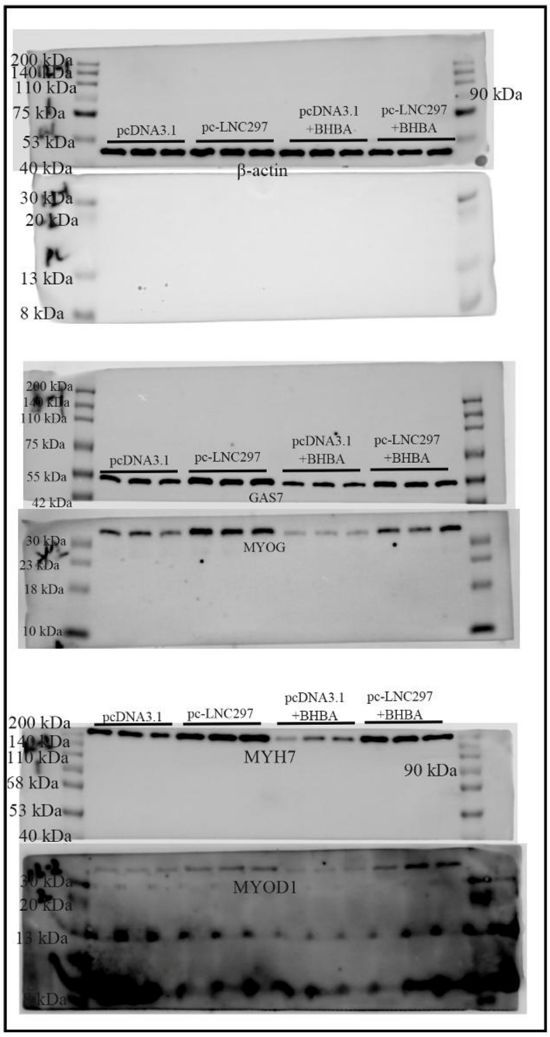

Original images

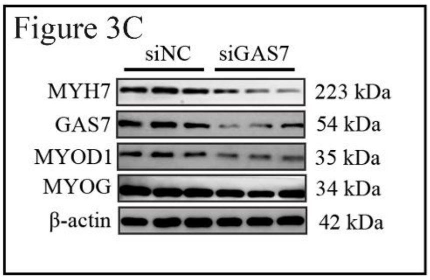

Figure in manuscript

Group	actin	MYOG	MYOD1	GAS7	MYH7
siNC	146468	217532	96357	115149	100356
siNC	168501	199059	99968	145472	110672
siNC	173075	186049	87776	125628	90571
siGAS7	158239	129567	39285	31213	49910
siGAS7	159951	113301	57589	47505	37832
siGAS7	165250	126771	42188	68173	28216

The original data of the gray values of each WB band in Figure 3C

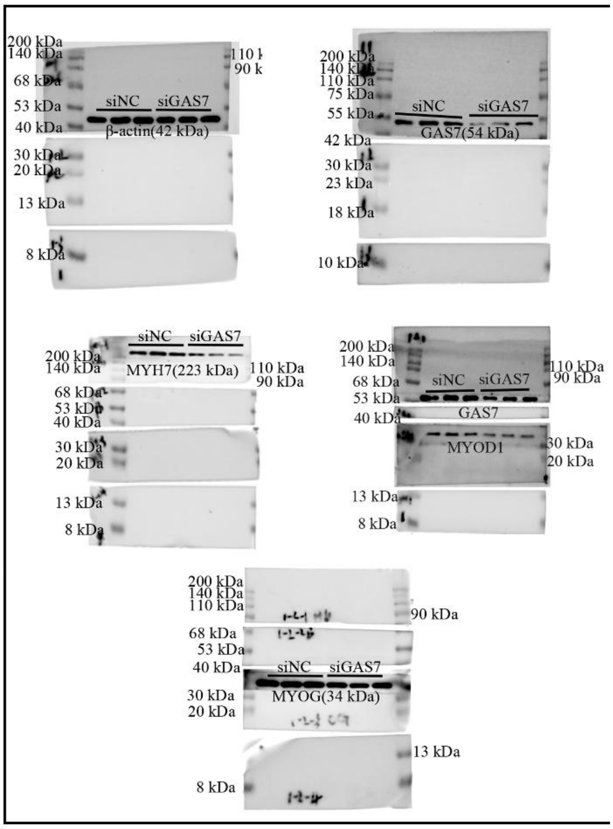

Original images

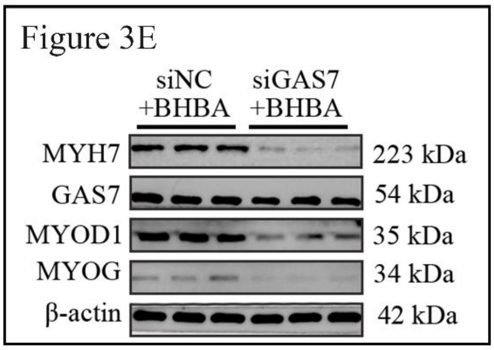

Figure in manuscript

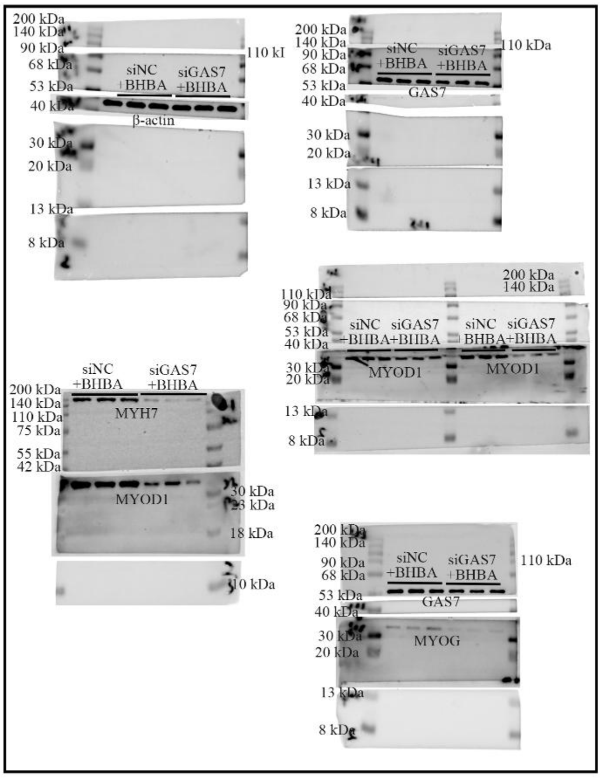

Original images

Group	actin	MYOG	MYOD1	GAS7	MYH7
siNC+BHBA	171821	60274	155336	153835	120129
siNC+BHBA	168702	59345	151004	140097	118326
siNC+BHBA	167371	86084	145457	142726	112104
siGAS7+BHBA	160835	26163	48619	72950	34251
siGAS7+BHBA	164129	25019	46647	94515	30060
siGAS7+BHBA	174402	27224	56075	88194	33203

The original data of the gray values of each WB band in Figure 3E

Figure 4E

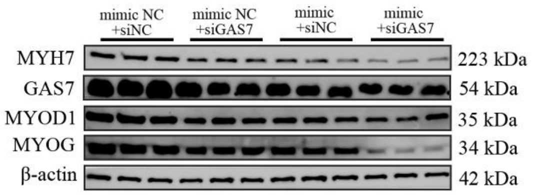

Figure in manuscript

Group	actin	MYOG	MYOD1	GAS7	MYH7
mimic NC+siNC	117962	312344	255209	357576	175455
mimic NC+siNC	105825	282944	240299	324760	164786
mimic NC+siNC	103117	269616	224034	338012	162212
mimic NC+siGAS7	95991	139781	124194	262072	68254
mimic NC+siGAS7	107314	143799	137316	263848	72761
mimic NC+siGAS7	101485	143048	127429	265803	67061
mimic+siNC	108635	187651	128263	258502	67606
mimic+siNC	114385	176316	127970	268140	89806
mimic+siNC	110990	170502	125361	260005	79347
mimic+siGAS7	100209	66648	88928	197286	39038
mimic+siGAS7	101680	54821	84154	196486	40179
mimic+siGAS7	101077	56530	101546	199878	42197

The original data of the gray values of each WB band in Figure 4E

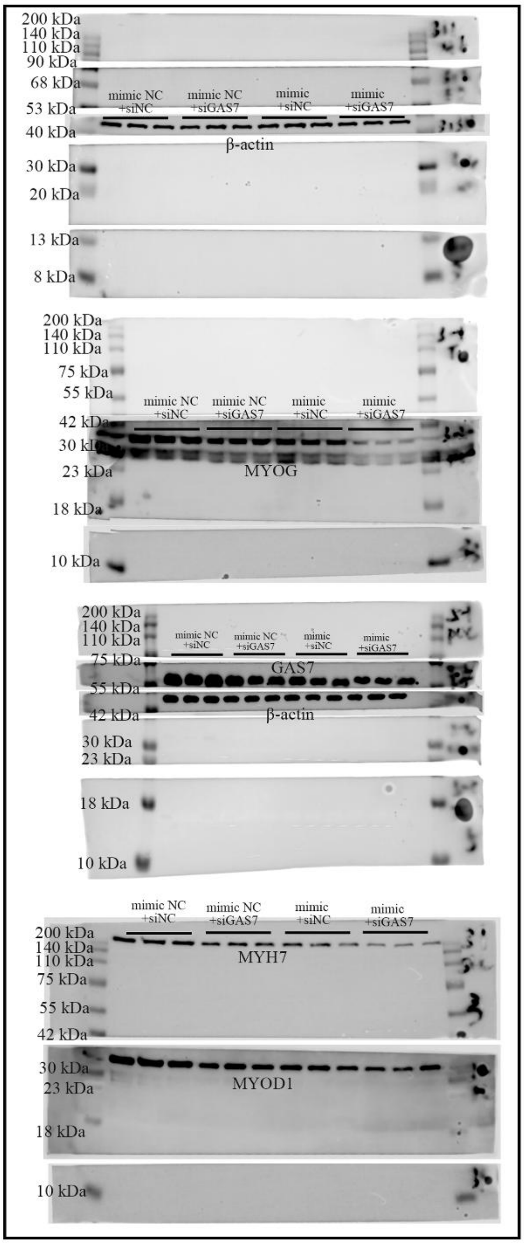

Original images

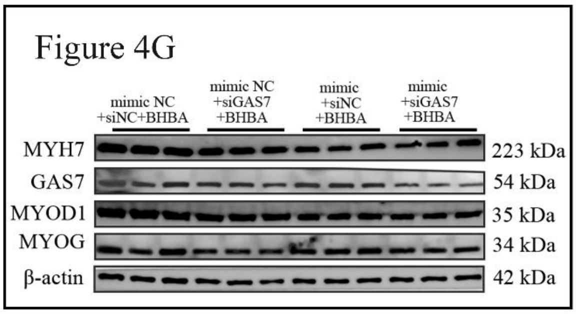

Figure in manuscript

Group	actin	MYOG	MYOD1	GAS7	MYH7
mimic NC+siNC+BHBA	117591	152399	258570	171168	241203
mimic NC+siNC+BHBA	112503	136687	254154	135545	228655
mimic NC+siNC+BHBA	119311	160838	239154	126659	225864
mimic NC+siGAS7+BHAB	106650	91709	169234	66236	155000
mimic NC+siGAS7+BHAB	107122	92527	167595	67355	149339
mimic NC+siGAS7+BHAB	95874	109183	163938	58599	144598
mimic+siNC+BHBA	103647	105038	155930	82656	132777
mimic+siNC+BHBA	115285	101262	154048	79928	121917
mimic+siNC+BHBA	113249	103289	156740	72355	128896
mimic+siGAS7+BHBA	113105	81079	99936	40957	119579
mimic+siGAS7+BHBA	114505	75796	103591	39111	127084
mimic+siGAS7+BHBA	124017	78355	101648	36868	106033

The original data of the gray values of each WB band in Figure 4G

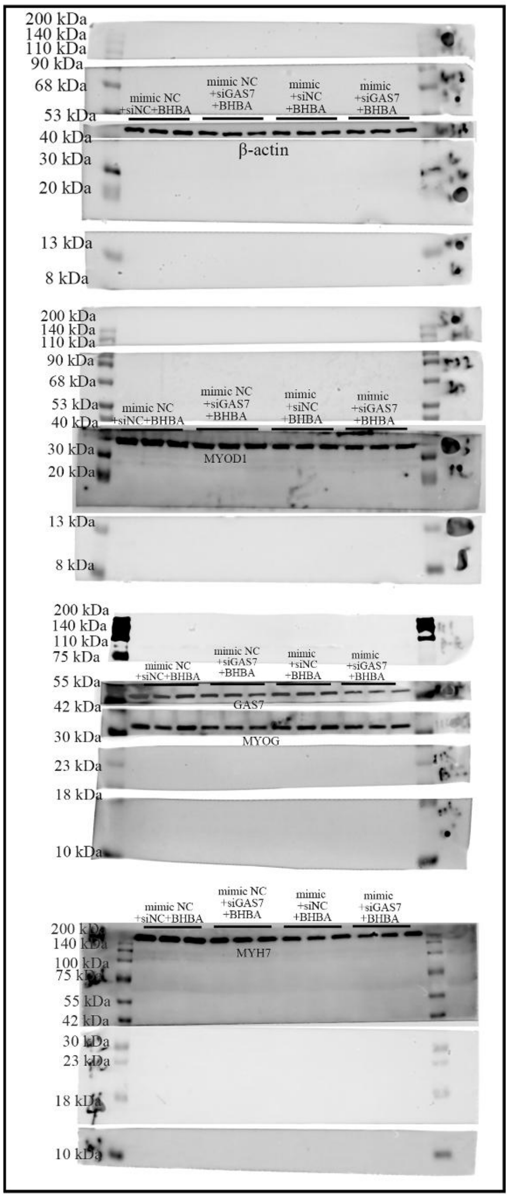

Original images

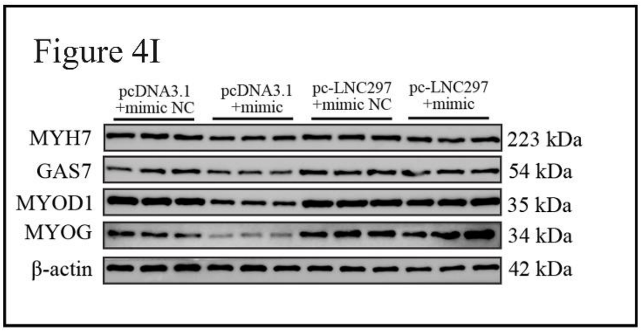

Figure in manuscript

Group	actin	MYOG	MYOD1	GAS7	MYH7
pcDNA3.1+mimic NC	141557	118826	156669	88484	108483
pcDNA3.1+mimic NC	146232	133937	147211	101106	105068
pcDNA3.1+mimic NC	154133	100126	141802	109546	106015
pcDNA3.1+mimic	137414	44339	84863	60033	71468
pcDNA3.1+mimic	142222	49777	83707	59526	70473
pcDNA3.1+mimic	130025	57077	79645	56082	73266
pc-LNC297+mimic NC	126201	160862	172347	124764	152477
pc-LNC297+mimic NC	125153	175261	197324	117463	147155
pc-LNC297+mimic NC	121465	161375	195427	158700	154542
pc-LNC297+mimic	113965	110576	136785	120297	97735
pc-LNC297+mimic	117275	144563	145875	116180	92179
pc-LNC297+mimic	126387	260852	155808	119628	104007

The original data of the gray values of each WB band in Figure 4I

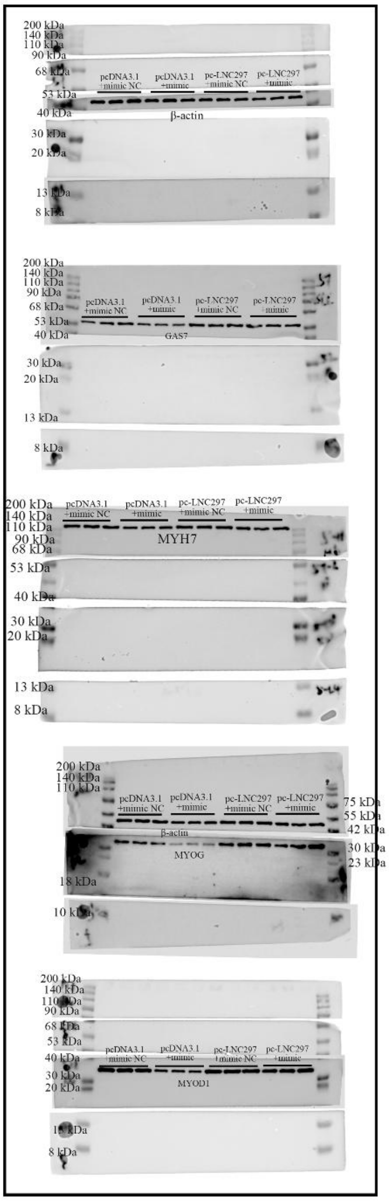

Original images

Figure 4K

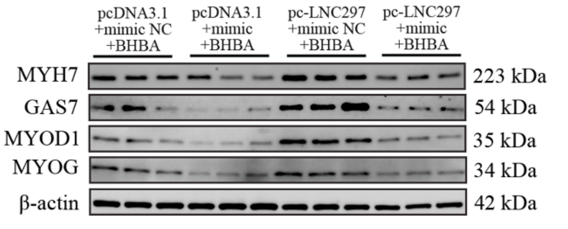

Figure in manuscript

Group	actin	MYOG	MYOD1	GAS7	MYH7
pcDNA3.1+mimic NC+BHBA	123351	119988	89228	108859	98985
pcDNA3.1+mimic NC+BHBA	119502	107307	82116	118525	90739
pcDNA3.1+mimic NC+BHBA	124575	91504	67454	75971	86553
pcDNA3.1+mimic+BHBA	120315	27582	21575	25979	57167
pcDNA3.1+mimic+BHBA	128880	34203	32541	26201	63216
pcDNA3.1+mimic+BHBA	132081	48080	44026	34736	41145
pc-LNC297+mimic NC+BHBA	115003	123384	128469	133361	151229
pc-LNC297+mimic NC+BHBA	115865	168843	125590	140208	138635
pc-LNC297+mimic NC+BHBA	107841	148577	113627	176165	130659
pc-LNC297+mimic+BHBA	103915	74733	63601	79839	73631
pc-LNC297+mimic+BHBA	96111	76326	63657	87355	80232
pc-LNC297+mimic+BHBA	103727	66522	76101	100379	91443

The original data of the gray values of each WB band in Figure 4K

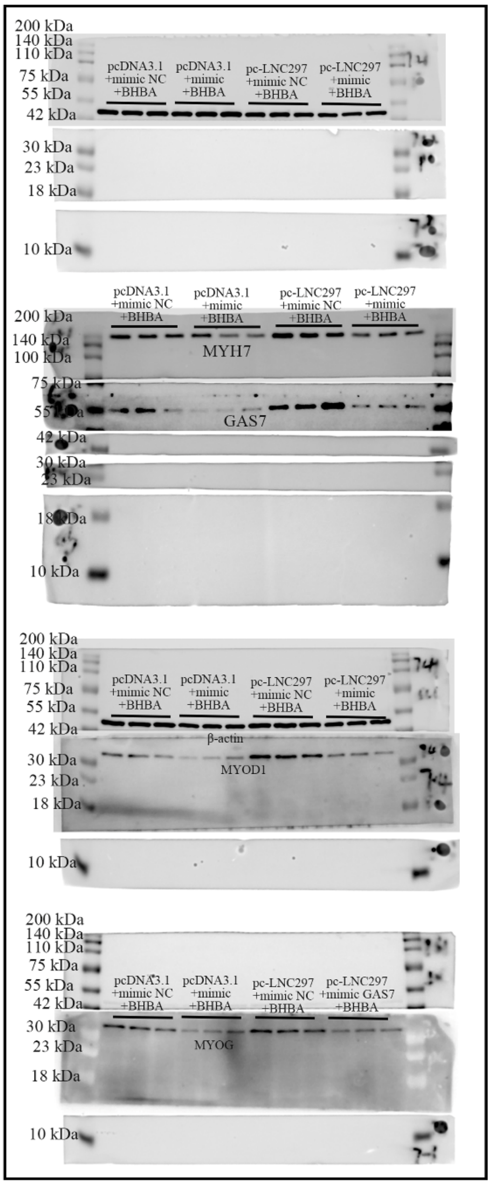

Original images
