# Supplementary material for: LNC297 promotes BMSCs differentiation and alleviates BHBA-induced inhibition through the miR-145/GAS7 axis
Source: PLoS One. 2026 Jul 22;21(7):e0354035. doi: 10.1371/journal.pone.0354035 (PMC13390936; doi:10.1371/journal.pone.0354035)
Supplement: Attachment — Submitted filename: Response to Reviewers.docx [file pone.0354035.s006.docx]

**Reviewer 1:**

Dear Reviewer:

On behalf of my co-authors, we thank you very much for giving us an opportunity to revise our manuscript, we appreciate you and reviewers very much for their positive and constructive comments and suggestions on our manuscript entitled “LNC297 Promotes BMSCs Differentiation and Alleviates BHBA-Induced Inhibition through the miR-145/GAS7 Axis” (**PONE-D-26-13971**). We have studied your and reviewer’s comments carefully and have made revision. The details are as follows:

**Comment #1:** **The first and second sentences in the abstract lack a clear logical connection; it is recommended to include relevant evidence to clarify the background.**

**Response:** Thank you for this helpful comment. We agree that the logical connection between the first two sentences was not sufficiently clear. To clarify the biological background, we have revised the opening of the Abstract by adding evidence that skeletal muscle is a major peripheral tissue involved in energy metabolism and is exposed to elevated circulating BHBA during ketosis. This revision better links ketosis-induced metabolic alterations with the potential role of BHBA in skeletal muscle regulation. The revised content is as follows:

**Comment #2:** **Regarding GAS7 knockdown with siRNA: the title of Section 3.3 (“GAS7 alleviates the inhibitory effect of BHBA”) in Figure 3 does not appear to be supported by the data. It would be more convincing to test whether over-expression of GAS7 can counteract the effects of BHBA. The same suggestion applies to the experiments shown in Figure 4; using over‑expression instead of siRNA would better support the authors’ conclusions.**

**Response：**We would like to thank the reviewer for their valuable comments on the experimental design. We understand the reviewer's concern regarding the title 'GAS7 alleviates the inhibitory effect of BHBA' and appreciate the suggestion to use GAS7 overexpression experiments to better validate our conclusions. The primary reason we chose to use siRNA to knock down GAS7 was to explore the effect of GAS7loss of function on BMSCs differentiation. The results show that when the expression of GAS7 in BMSCs is reduced, the expression of differentiation marker genes MYH7, MYOD1, and MYOG is downregulated, suggesting that GAS7 promotes BMSC differentiation. Some of the results in Section 3.2 demonstrate that BHBA inhibits BMSC differentiation, and when Battement is added while lowering GAS7 expression, this inhibition is further exacerbated. Therefore, we hypothesize that GAS7 alleviates the inhibitory effect of BHBA. Of course, these data may be better explained through a rescue experiment using overexpression. We are grateful for the reviewer’feedback on the experimental design flaws in this study. Finally, we would like to express our sincere gratitude to the reviewer for the valuable suggestions, which will provide precious insights for the design.

**Comment #3:"Replace the differentiation medium with an induction medium containing 4.8 mM BHBA and continue culturing for 12 hours." Why is a 12-hour treatment duration used?**

**Response**: We sincerely thank the reviewer for the valuable comments. Previous studies have shown that treatment of C_2_C_12_ cells with sodium β-hydroxybutyrate for 24 hours can promote myoblast proliferation and differentiation through energy metabolism and the GPR109A-mediated Ca²⁺-NFAT signaling pathway. However, in our study, we used β-hydroxybutyric acid (BHBA) and BMSCs, which differ from both the compound and the cell type used in prior work. Therefore, we shortened the treatment duration accordingly. In our experiments, a 12-hour treatment was sufficient to observe initial cellular responses and changes in the expression of differentiation markers. Although this duration may not fully support complete satellite cell proliferation and differentiation, it allows a subset of cells to enter the differentiation process and provides meaningful data regarding molecular marker dynamics. In our experimental design, the 12-hour treatment was intended as a preliminary induction period to assess early cellular responses within a short timeframe, and to inform future experiments involving longer treatment durations. We will clarify this point further in the Methods section, emphasizing that the 12-hour treatment time is a rational choice based on previous literature and preliminary results. Finally, we would like to express our gratitude to the reviewers for their valuable comments. The limitations of this research are also discussed in the article. Additionally, the methodology section (2.9) of the article also cites relevant literature to support the reasons for using this processing time.

**Comment #4: Line 225 "ofmiR-145" should be changed to "of miR-145".**

**Response**: Thank you very much for the reviewers' comments. The revisions have been made in the manuscript.

**Comment #5: On line 244, "overexpressing LNC297" should be changed to "overexpressing LNC297", and on line 245, "LNC297promotes" should be changed to "LNC297 promotes". All relevant issues should be checked throughout the text.**

**Response**: Thank you very much for the reviewers' comments. The revisions have been made in the manuscript.

**Comment #6: The presence or absence of spaces between the reference citations and the main text needs to be uniformly standardized throughout the entire document.**

**Response**: Thank you very much for the reviewers' comments. I have made the necessary revisions as per the journal's requirements.
