# Supplementary material for: LNC297 promotes BMSCs differentiation and alleviates BHBA-induced inhibition through the miR-145/GAS7 axis
Source: PLoS One. 2026 Jul 22;21(7):e0354035. doi: 10.1371/journal.pone.0354035 (PMC13390936; doi:10.1371/journal.pone.0354035)
Supplement: Peer review history

# Peer review: Decision Letter 0

**DOI:** 10.1371/journal.pone.0354035.r001

**Authors:** Gary Stein

25 Jun 2026

PONE-D-26-13971

LNC297 Promotes BMSCs Differentiation and Alleviates BHBA-Induced Inhibition through the miR-145/GAS7 Axis

PLOS One

Dear Dr. Lai,

Thank you for submitting your manuscript to PLOS ONE. After careful consideration, we feel that it has merit but does not fully meet PLOS ONE’s publication criteria as it currently stands. Therefore, we invite you to submit a revised version of the manuscript that addresses the points raised during the review process.

Please submit your revised manuscript by Aug 09 2026 11:59PM. If you will need more time than this to complete your revisions, please reply to this message or contact the journal office at plosone@plos.org. When you're ready to submit your revision, log on to https://www.editorialmanager.com/pone/ and select the 'Submissions Needing Revision' folder to locate your manuscript file.

Please include the following items when submitting your revised manuscript:

- A letter that responds to each point raised by the academic editor and reviewer(s). You should upload this letter as a separate file labeled 'Response to Reviewers'.
- A marked-up copy of your manuscript that highlights changes made to the original version. You should upload this as a separate file labeled 'Revised Manuscript with Track Changes'.
- An unmarked version of your revised paper without tracked changes. You should upload this as a separate file labeled 'Manuscript'.

If you would like to make changes to your financial disclosure, please include your updated statement in your cover letter. Guidelines for resubmitting your figure files are available below the reviewer comments at the end of this letter.

If applicable, we recommend that you deposit your laboratory protocols in protocols.io to enhance the reproducibility of your results. Protocols.io assigns your protocol its own identifier (DOI) so that it can be cited independently in the future. For instructions see: https://journals.plos.org/plosone/s/submission-guidelines#loc-laboratory-protocols. Additionally, PLOS ONE offers an option for publishing peer-reviewed Lab Protocol articles, which describe protocols hosted on protocols.io. Read more information on sharing protocols at https://plos.org/protocols?utm_medium=editorial-email&utm_source=authorletters&utm_campaign=protocols.

As the corresponding author, your ORCID iD is verified in the submission system and will appear in the published article. PLOS supports the use of ORCID, and we encourage all coauthors to register for an ORCID iD and use it as well. Please encourage your coauthors to verify their ORCID iD within the submission system before final acceptance, as unverified ORCID iDs will not appear in the published article. *Only*  the individual author can complete the verification step; PLOS staff *cannot*  verify ORCID iDs on behalf of authors.

We look forward to receiving your revised manuscript.

Kind regards,

Gary S. Stein

Academic Editor

PLOS One

Journal Requirements:

When submitting your revision, we need you to address these additional requirements.

1. Please ensure that your manuscript meets PLOS ONE's style requirements, including those for file naming. The PLOS ONE style templates can be found at

https://journals.plos.org/plosone/s/file?id=wjVg/PLOSOne_formatting_sample_main_body.pdf and

https://journals.plos.org/plosone/s/file?id=ba62/PLOSOne_formatting_sample_title_authors_affiliations.pdf.

2. Thank you for stating the following financial disclosure:

“This research was funded by the Sichuan innovation team of national modern agricultural industry technology system, grant number SCCXTD-2026-13; Sichuan key research and development project on innovative breeding materials, methods, and new breeds of high-quality cattle, Key R&D Program of Sichuan Province, grant number 2021YFYZ0001; Major Livestock and Poultry Germplasm Re-sources (Breeding Breakthrough Project), Key R&D Program of Sichuan Province, grant number 2021YFYZ0007. “

Please state what role the funders took in the study.  If the funders had no role, please state: “The funders had no role in study design, data collection and analysis, decision to publish, or preparation of the manuscript.”

If this statement is not correct you must amend it as needed.

Please include this amended Role of Funder statement in your cover letter; we will change the online submission form on your behalf.

3. PLOS ONE now requires that authors provide the original uncropped and unadjusted images underlying all blot or gel results reported in a submission’s figures or Supporting Information files. This policy and the journal’s other requirements for blot/gel reporting and figure preparation are described in detail at https://journals.plos.org/plosone/s/figures#loc-blot-and-gel-reporting-requirements and https://journals.plos.org/plosone/s/figures#loc-preparing-figures-from-image-files. When you submit your revised manuscript, please ensure that your figures adhere fully to these guidelines and provide the original underlying images for all blot or gel data reported in your submission. See the following link for instructions on providing the original image data: https://journals.plos.org/plosone/s/figures#loc-original-images-for-blots-and-gels.

In your cover letter, please note whether your blot/gel image data are in Supporting Information or posted at a public data repository, provide the repository URL if relevant, and provide specific details as to which raw blot/gel images, if any, are not available. Email us at plosone@plos.org if you have any questions.

4. Please be informed that funding information should not appear in the Acknowledgments section or other areas of your manuscript. We will only publish funding information present in the Funding Statement section of the online submission form. Please remove any funding-related text from the manuscript.

5. Thank you for stating the following in the Competing Interests section:

“The authors have declared that no competing interests exist. “

We note that one or more of the authors are employed by a commercial company: Hong Feng Dairy Cow Breeding Co., Ltd

a.        Please provide an amended Funding Statement declaring this commercial affiliation, as well as a statement regarding the Role of Funders in your study. If the funding organization did not play a role in the study design, data collection and analysis, decision to publish, or preparation of the manuscript and only provided financial support in the form of authors' salaries and/or research materials, please review your statements relating to the author contributions, and ensure you have specifically and accurately indicated the role(s) that these authors had in your study. You can update author roles in the Author Contributions section of the online submission form.

Please also include the following statement within your amended Funding Statement.

“The funder provided support in the form of salaries for authors [insert relevant initials], but did not have any additional role in the study design, data collection and analysis, decision to publish, or preparation of the manuscript. The specific roles of these authors are articulated in the ‘author contributions’ section.”

If your commercial affiliation did play a role in your study, please state and explain this role within your updated Funding Statement.

b. Please also provide an updated Competing Interests Statement declaring this commercial affiliation along with any other relevant declarations relating to employment, consultancy, patents, products in development, or marketed products, etc.

Within your Competing Interests Statement, please confirm that this commercial affiliation does not alter your adherence to all PLOS ONE policies on sharing data and materials by including the following statement: “This does not alter our adherence to  PLOS ONE policies on sharing data and materials.” (as detailed online in our guide for authors http://journals.plos.org/plosone/s/competing-interests) . If this adherence statement is not accurate and  there are restrictions on sharing of data and/or materials, please state these. Please note that we cannot proceed with consideration of your article until this information has been declared.

Please include both an updated Funding Statement and Competing Interests Statement in your cover letter. We will change the online submission form on your behalf.

6. Please include captions for your Supporting Information files at the end of your manuscript, and update any in-text citations to match accordingly. Please see our Supporting Information guidelines for more information: http://journals.plos.org/plosone/s/supporting-information.

7. If the reviewer comments include a recommendation to cite specific previously published works, please review and evaluate these publications to determine whether they are relevant and should be cited. There is no requirement to cite these works unless the editor has indicated otherwise.

8. Please review your reference list to ensure that it is complete and correct. If you have cited papers that have been retracted, please include the rationale for doing so in the manuscript text, or remove these references and replace them with relevant current references. Any changes to the reference list should be mentioned in the rebuttal letter that accompanies your revised manuscript. If you need to cite a retracted article, indicate the article’s retracted status in the References list and also include a citation and full reference for the retraction notice.

[Note: HTML markup is below. Please do not edit.]

Reviewers' comments:

Reviewer's Responses to Questions

**Comments to the Author**

1. Is the manuscript technically sound, and do the data support the conclusions?

The manuscript must describe a technically sound piece of scientific research with data that supports the conclusions. Experiments must have been conducted rigorously, with appropriate controls, replication, and sample sizes. The conclusions must be drawn appropriately based on the data presented.

Reviewer #1: Yes

**********

2. Has the statistical analysis been performed appropriately and rigorously?

Reviewer #1: Yes

**********

3. Have the authors made all data underlying the findings in their manuscript fully available?

The PLOS Data policy requires authors to make all data underlying the findings described in their manuscript fully available without restriction, with rare exception (please refer to the Data Availability Statement in the manuscript PDF file). The data should be provided as part of the manuscript or its supporting information, or deposited to a public repository. For example, in addition to summary statistics, the data points behind means, medians and variance measures should be available. If there are restrictions on publicly sharing data—e.g. participant privacy or use of data from a third party—those must be specified.

Reviewer #1: Yes

**********

4. Is the manuscript presented in an intelligible fashion and written in standard English?

PLOS ONE does not copyedit accepted manuscripts, so the language in submitted articles must be clear, correct, and unambiguous. Any typographical or grammatical errors should be corrected at revision, so please note any specific errors here.

Reviewer #1: Yes

**********

5. Review Comments to the Author

Please use the space provided to explain your answers to the questions above. You may also include additional comments for the author, including concerns about dual publication, research ethics, or publication ethics. (Please upload your review as an attachment if it exceeds 20,000 characters)

Reviewer #1: When ketosis occurs, dairy bovine muscle fibers can become damaged. It was unclear whether β‑hydroxybutyrate (BHBA), a type of ketone body, is involved in this phenomenon. This study examined the effects of BHBA on muscle fiber function and its molecular mechanisms in vitro. The results showed that treating bovine muscle satellite cells (BMSCs) with BHBA suppressed myogenic differentiation. Furthermore, the authors previously identified LNC297 and miR‑145 as having altered expression during instances of muscle fiber damage in ketosis bovines; similar changes were observed in this study. Subsequent database analyses and in vitro experiments involving miRNA mimics, siRNAs, and overexpression constructs demonstrated that: miR‑145 enhances the inhibitory effect of BHBA on myogenic differentiation; miR‑145 suppresses the expression of GAS7, a gene involved in muscle fiber growth and maintenance; LNC297 can negate the effects of BHBA by potentially binding to and inhibiting miR‑145. The data presented are clear, shedding light on aspects of BHBA’s effect and its underlying molecular mechanism. Beyond addressing issues associated with ketosis in dairy bovines, this study may also have implications for resolving human ketosis conditions in the future. However, there are a few issues in the manuscript that need to be addressed:

1.The first and second sentences in the abstract lack a clear logical connection; it is recommended to include relevant evidence to clarify the background.

2.Regarding GAS7 knockdown with siRNA: the title of Section 3.3 (“GAS7 alleviates the inhibitory effect of BHBA”) in Figure 3 does not appear to be supported by the data. It would be more convincing to test whether over-expression of GAS7 can counteract the effects of BHBA. The same suggestion applies to the experiments shown in Figure 4; using over‑expression instead of siRNA would better support the authors’ conclusions.

3."Replace the differentiation medium with an induction medium containing 4.8 mM BHBA and continue culturing for 12 hours." Why is a 12-hour treatment duration used?

4.Line 225 "ofmiR-145" should be changed to "of miR-145".

5.On line 244, "overexpressing LNC297" should be changed to "overexpressing LNC297", and on line 245, "LNC297promotes" should be changed to "LNC297 promotes". All relevant issues should be checked throughout the text.

6.The presence or absence of spaces between the reference citations and the main text needs to be uniformly standardized throughout the entire document.

**********

6. PLOS authors have the option to publish the peer review history of their article (what does this mean?). If published, this will include your full peer review and any attached files.

If you choose “no”, your identity will remain anonymous but your review may still be made public.

**Do you want your identity to be public for this peer review?**  For information about this choice, including consent withdrawal, please see our Privacy Policy.

Reviewer #1: No

**********

[NOTE: If reviewer comments were submitted as an attachment file, they will be attached to this email and accessible via the submission site. Please log into your account, locate the manuscript record, and check for the action link "View Attachments". If this link does not appear, there are no attachment files.]

To ensure your figures meet our technical requirements, please review our figure guidelines: https://journals.plos.org/plosone/s/figures

You may also use PLOS’s free figure tool, NAAS, to help you prepare publication quality figures: https://journals.plos.org/plosone/s/figures#loc-tools-for-figure-preparation.

NAAS will assess whether your figures meet our technical requirements by comparing each figure against our figure specifications.

# Author comment: Author response to Decision Letter 1

**DOI:** 10.1371/journal.pone.0354035.r002

1 Jul 2026

Reviewer 1:

Dear Reviewer:

On behalf of my co-authors, we thank you very much for giving us an opportunity to revise our manuscript, we appreciate you and reviewers very much for their positive and constructive comments and suggestions on our manuscript entitled “LNC297 Promotes BMSCs Differentiation and Alleviates BHBA-Induced Inhibition through the miR-145/GAS7 Axis” (PONE-D-26-13971). We have studied your and reviewer’s comments carefully and have made revision. The details are as follows:

Comment #1: The first and second sentences in the abstract lack a clear logical connection; it is recommended to include relevant evidence to clarify the background.

Response: Thank you for this helpful comment. We agree that the logical connection between the first two sentences was not sufficiently clear. To clarify the biological background, we have revised the opening of the Abstract by adding evidence that skeletal muscle is a major peripheral tissue involved in energy metabolism and is exposed to elevated circulating BHBA during ketosis. This revision better links ketosis-induced metabolic alterations with the potential role of BHBA in skeletal muscle regulation. The revised content is as follows:

Comment #2: Regarding GAS7 knockdown with siRNA: the title of Section 3.3 (“GAS7 alleviates the inhibitory effect of BHBA”) in Figure 3 does not appear to be supported by the data. It would be more convincing to test whether over-expression of GAS7 can counteract the effects of BHBA. The same suggestion applies to the experiments shown in Figure 4; using over‑expression instead of siRNA would better support the authors’ conclusions.

Response�We would like to thank the reviewer for their valuable comments on the experimental design. We understand the reviewer's concern regarding the title 'GAS7 alleviates the inhibitory effect of BHBA' and appreciate the suggestion to use GAS7 overexpression experiments to better validate our conclusions. The primary reason we chose to use siRNA to knock down GAS7 was to explore the effect of GAS7loss of function on BMSCs differentiation. The results show that when the expression of GAS7 in BMSCs is reduced, the expression of differentiation marker genes MYH7, MYOD1, and MYOG is downregulated, suggesting that GAS7 promotes BMSC differentiation. Some of the results in Section 3.2 demonstrate that BHBA inhibits BMSC differentiation, and when Battement is added while lowering GAS7 expression, this inhibition is further exacerbated. Therefore, we hypothesize that GAS7 alleviates the inhibitory effect of BHBA. Of course, these data may be better explained through a rescue experiment using overexpression. We are grateful for the reviewer’feedback on the experimental design flaws in this study. Finally, we would like to express our sincere gratitude to the reviewer for the valuable suggestions, which will provide precious insights for the design.

Comment #3:"Replace the differentiation medium with an induction medium containing 4.8 mM BHBA and continue culturing for 12 hours." Why is a 12-hour treatment duration used?

Response: We sincerely thank the reviewer for the valuable comments. Previous studies have shown that treatment of C2C12 cells with sodium β-hydroxybutyrate for 24 hours can promote myoblast proliferation and differentiation through energy metabolism and the GPR109A-mediated Ca²⁺-NFAT signaling pathway. However, in our study, we used β-hydroxybutyric acid (BHBA) and BMSCs, which differ from both the compound and the cell type used in prior work. Therefore, we shortened the treatment duration accordingly. In our experiments, a 12-hour treatment was sufficient to observe initial cellular responses and changes in the expression of differentiation markers. Although this duration may not fully support complete satellite cell proliferation and differentiation, it allows a subset of cells to enter the differentiation process and provides meaningful data regarding molecular marker dynamics. In our experimental design, the 12-hour treatment was intended as a preliminary induction period to assess early cellular responses within a short timeframe, and to inform future experiments involving longer treatment durations. We will clarify this point further in the Methods section, emphasizing that the 12-hour treatment time is a rational choice based on previous literature and preliminary results. Finally, we would like to express our gratitude to the reviewers for their valuable comments. The limitations of this research are also discussed in the article. Additionally, the methodology section (2.9) of the article also cites relevant literature to support the reasons for using this processing time.

Comment #4: Line 225 "ofmiR-145" should be changed to "of miR-145".

Response: Thank you very much for the reviewers' comments. The revisions have been made in the manuscript.

Comment #5: On line 244, "overexpressing LNC297" should be changed to "overexpressing LNC297", and on line 245, "LNC297promotes" should be changed to "LNC297 promotes". All relevant issues should be checked throughout the text.

Response: Thank you very much for the reviewers' comments. The revisions have been made in the manuscript.

Comment #6: The presence or absence of spaces between the reference citations and the main text needs to be uniformly standardized throughout the entire document.

Response: Thank you very much for the reviewers' comments. I have made the necessary revisions as per the journal's requirements.

Attachment Submitted filename: Response to Reviewers.docx

# Editor report: Decision Letter 1

**DOI:** 10.1371/journal.pone.0354035.r003

**Authors:** Gary Stein

2 Jul 2026

LNC297 Promotes BMSCs Differentiation and Alleviates BHBA-Induced Inhibition through the miR-145/GAS7 Axis

PONE-D-26-13971R1

Dear Dr. Lai,

We’re pleased to inform you that your manuscript has been judged scientifically suitable for publication and will be formally accepted for publication once it meets all outstanding technical requirements.

Within one week, you’ll receive an e-mail detailing the required amendments. When these have been addressed, you’ll receive a formal acceptance letter and your manuscript will be scheduled for publication.

An invoice will be generated when your article is formally accepted. Please note, if your institution has a publishing partnership with PLOS and your article meets the relevant criteria, all or part of your publication costs will be covered. Please make sure your user information is up-to-date by logging into Editorial Manager at Editorial Manager® and clicking the ‘Update My Information' link at the top of the page. For questions related to billing, please contact billing support.

If your institution or institutions have a press office, please notify them about your upcoming paper to help maximize its impact. If they’ll be preparing press materials, please inform our press team as soon as possible -- no later than 48 hours after receiving the formal acceptance. Your manuscript will remain under strict press embargo until 2 pm Eastern Time on the date of publication. For more information, please contact onepress@plos.org.

Kind regards,

Gary S. Stein

Academic Editor

PLOS One

# Editor report: Acceptance letter

**DOI:** 10.1371/journal.pone.0354035.r004

**Authors:** Gary Stein

PONE-D-26-13971R1

PLOS One

Dear Dr. Lai,

I'm pleased to inform you that your manuscript has been deemed suitable for publication in PLOS One. Congratulations! Your manuscript is now being handed over to our production team.

At this stage, our production department will prepare your paper for publication. This includes ensuring the following:

* All references, tables, and figures are properly cited

* All relevant supporting information is included in the manuscript submission,

* There are no issues that prevent the paper from being properly typeset

You will receive further instructions from the production team, including instructions on how to review your proof when it is ready. Please keep in mind that we are working through a large volume of accepted articles, so please give us a few days to review your paper and let you know the next and final steps.

Lastly, if your institution or institutions have a press office, please let them know about your upcoming paper now to help maximize its impact. If they'll be preparing press materials, please inform our press team within the next 48 hours. Your manuscript will remain under strict press embargo until 2 pm Eastern Time on the date of publication. For more information, please contact onepress@plos.org.

You will receive an invoice from PLOS for your publication fee after your manuscript has reached the completed accept phase. If you receive an email requesting payment before acceptance or for any other service, this may be a phishing scheme. Learn how to identify phishing emails and protect your accounts at https://explore.plos.org/phishing.

If we can help with anything else, please email us at customercare@plos.org.

Thank you for submitting your work to PLOS ONE and supporting open access.

Kind regards,

PLOS ONE Editorial Office Staff

on behalf of

Dr. Gary S. Stein

Academic Editor

PLOS One
